# VEZF1 inhibits ovarian cancer cell ferroptosis and acts as an oncogene via the miR-545-3p/PLAG1 axis

**DOI:** 10.1186/s41065-026-00672-z

**Published:** 2026-04-13

**Authors:** Wei Shi, Weiwei Feng, Jing Wan, Xiao-Ping Wan

**Affiliations:** 1 Shanghai Key Laboratory of Maternal Fetal Medicine, Shanghai Institute of Maternal-Fetal Medicine and Gynecologic Oncology, Shanghai First Maternity and Infant Hospital, School of Medicine, Tongji University, No.550 Hu’nan Road, Shanghai, 200092 China; 2 Department of Gynaecology and Obstetrics, School of Medicine, The International Peace Maternity and Child Health Hospital, Shanghai Jiao Tong University, Shanghai, 200030 China

**Keywords:** Ovarian cancer, High glucose, Ferroptosis, VEZF1, miR-545-3p, PLAG1

## Abstract

**Background:**

Ovarian cancer (OC) is a fatal carcinoma for women. This study attempts to explore the role of vascular endothelial zinc finger 1 (VEZF1) in OC cell ferroptosis, thereby finding a new target for OC treatment.

**Methods:**

Expressions of VEZF1, miR-545-3p and PLAG1 in HOSE cell line and OC cell lines were determined by RT-qPCR and western blot analysis. After VEZF1 was silenced in cells, cell proliferation was examined, contents of ROS, MDA, Fe^2+^, GSH were detected, and expressions of ACSL4 and GPX4 were tested. Afterwards, the binding relation between VEZF1 and miR-545-3p and between miR-545-3p and PLAG1 were verified. Functional rescue assays were formulated to validate the role of miR-545-3p knockdown or PLAG1 overexpression in OC cell ferroptosis.

**Results:**

VEZF1 was overexpressed in OC, and VEZF1 silencing reduced cell proliferation, elevated levels of ROS, MDA, Fe^2+^, inactivated levels of GSH and GPX4, and enhanced ACSL4 expression. Functionally, VEZF1 transcriptionally inhibited miR-545-3p, and miR-545-3p targeted and inhibited PLAG1. miR-545-3p knockdown or PLAG1 overexpression could reverse the effect of VEZF1 silencing on OC cell ferroptosis.

**Conclusion:**

VEZF1 was overexpressed in OC and it limited OC cell ferroptosis by transcriptionally inhibiting miR-545-3p and elevating PLAG1 expression.

**Supplementary Information:**

The online version contains supplementary material available at 10.1186/s41065-026-00672-z.

## Introduction

Ovarian cancer (OC) remains one of the most lethal gynecological malignancies, with high recurrence rates and poor overall survival due to its insidious onset and frequent diagnosis at advanced stages [1]. Family history of gynecological diseases, excessive hormone administration, diabetes, unhealthy lifestyle, and overweight are major reasons for OC development [2]. Although cytoreductive surgery, platinum-based chemotherapy, and targeted therapies (e.g., PARP inhibitors and anti-angiogenic agents) are standard treatments, drug resistance and toxicity severely limit their long-term efficacy [3]. Elucidating the molecular mechanisms driving OC progression is therefore essential for identifying novel therapeutic strategies. Ferroptosis, an iron-dependent form of regulated cell death characterized by lethal lipid peroxidation and iron overload, has emerged as a promising mechanism for inducing cancer cell death and overcoming therapy resistance [4]. Key regulators of ferroptosis include glutathione peroxidase 4 (GPX4), a selenoprotein that utilizes glutathione (GSH) to reduce lipid hydroperoxides, thereby inhibiting ferroptosis and maintaining cellular redox balance [5]. The cystine/glutamate antiporter system Xc-, primarily mediated by SLC7A11, facilitates cystine uptake for GSH synthesis and serves as a critical negative regulator of ferroptosis in cancer cells [6]. Additionally, acyl-CoA synthetase long-chain family member 4 (ACSL4) promotes ferroptosis by incorporating polyunsaturated fatty acids into membrane phospholipids, making cells more susceptible to lipid peroxidation [7]. Ferroptosis can modulate molecular homeostasis, gene expression, cellular response to therapy, and prognostic results of diverse carcinomas, including OC [8]. Ferrostatin-1 (Fer-1) is a synthetic radical-trapping antioxidant that prevents the accumulation of lipid peroxidation and blocks ferroptosis, functioning as an important ferroptosis inhibitor for verifying the occurrence of ferroptosis in OC [9–11]. Under this circumstance, biomarkers targeting OC cell ferroptosis are explored in our research.

Transcription factors are central regulators of cancer development, often interacting with microRNAs (miRNAs) to control downstream targets involved in tumor progression [12, 13]. Vascular endothelial zinc finger 1 (VEZF1), a zinc finger transcription factor, is overexpressed in OC and promotes malignant phenotypes, including cell proliferation, migration, and invasion [14–16]. VEZF1 negatively regulates specific miRNAs in various contexts, such as miR-382-5p in osteosarcoma [17] and miR-188-3p in podocyte ferroptosis [18]. In OC, we identified miR-545-3p as a downstream target of VEZF1. Notably, miR-545-3p knockdown is correlated with sabotaged ferroptosis in gastric cancer [19]. Bioinformatics analysis further revealed pleomorphic adenoma gene 1 (PLAG1) as a key target of miR-545-3p. PLAG1 expression is activated in OC, which is linked to the encouraged OC cell malignant behaviors and retarded drug sensitivity [20]. PLAG1 deprives hepatocellular carcinoma of cell ferroptosis to reduce clinic efficiency of clinic treatment [21]. Collectively, functional assays are conducted to elaborate the exact mechanism of VEZF1 in OC cell ferroptosis via the manipulation of the miR-545-3p/PLAG1 pathway, thus extending the understanding of OC attenuation.

## Materials and methods

### Cell culture

Normal human ovarian surface epithelium (HOSE) cell line and OC cell lines (SK-OV-3, OVCAR-3 and A2780) (all from Chinese Academy of Sciences Cell Bank, Shanghai, China) were cultured in Dulbecco’s modified Eagle’s medium (Invitrogen, Carlsbad, USA) containing 10% fetal bovine serum, 100 U/mL penicillin and 100 mg/L streptomycin in an incubator with 5% CO_2_ at 37℃.

### Cell treatment

SK-OV-3 or A2780 cells (1 × 10^5^cells/well) were seeded into 24-well plates and incubated overnight, and then transfected with small interfering (si)-VEZF1 (si-VEZF1#1, si-VEZF1#2b or si-VEZF1#3), si-negative control (NC), miR-545-3p inhibitor (miR-inhibitor), inhibitor NC, overexpression (oe)-PLAG1, or oe-NC (all from RiboBio Co., Ltd., Guangzhou, Guangdong, China) using Lipofectamine 2000 (Invitrogen). The detailed procedures were as follows: 20 pmol siRNA oligomers or 0.8 µg DNA were diluted in 50 µL serum-free Opti-MEM I reduced serum medium, followed by gentle mixing. Prior to use, Lipofectamine 2000 was gently vortexed; subsequently, 1 µL was aliquoted and diluted in 50 µL Opti-MEM I medium, then incubated at room temperature for 5 min. Following the incubation, the diluted DNA (or oligomers) was mixed with the diluted Lipofectamine 2000, gently homogenized, and incubated at room temperature for 20 min. The resulting complexes were added to each well containing cells and culture medium, with gentle agitation to ensure uniform mixing. At 24 h post-transfection, cells were passaged into fresh growth medium. Afterwards, transfection efficiency was evaluated by reverse transcription quantitative polymerase chain reaction (RT-qPCR). In the Fer-1 group, cells were preincubated with 1 mM Fer-1 for 1 h prior to transfection with si-VEZF1#1, with dimethyl sulfoxide (DMSO) serving as the negative control.

### Cell counting kit-8 (CCK-8) method

SK-OV-3 or A2780 cell proliferation was assessed by CCK-8 method (Keygen Biotech CO., Ltd, Nanjing, Jiangsu, China). Briefly, SK-OV-3 or A2780 cells (1 × 10^4^ cells/well) were seeded into 96-well plates and cultured in a humidified incubator at 37℃ for 24 h, with 10 µL CCK-8 solution added into each well for the following 1 h-culture in the humidified incubator at 37℃. The optical density (OD) value at 450 nm was analyzed using a microplate reader (Bio-Rad Laboratories, Hercules, CA, USA). Independent experiments were repeated 3 times.

### RT-qPCR

Trizol Reagent (Invitrogen) was appointed to extract total RNA from cells, and 2 µg RNA was applied for RT-qPCR using Qiagen OneStep RT-PCR kit (Qiagen Benelux BV, Venlo, The Netherlands). Gene expressions were quantified using TaqMan miRNA assay kit (Applied Biosystems; Thermo Fisher Scientific Inc., Waltham, MA, USA). Glyceraldehyde-3-phosphate dehydrogenase (GAPDH) and U6 acted as the internal reference [22]. 2^−ΔΔCT^ method was used to quantify relative expression of genes [23]. Primer sequences are listed on Table 1.

**Table 1:** Primers sequence of RT-qPCR

Genes	Sequences (5’-3’)
VEZF1	F: GACCGCGTTCCTGTTCCA
	R: GTCCCTGCTTCTTGGGTGAA
miR-545-3p	F: TCGGCAGGCCCAGCCT
	R: CTCAACTGGTGTCGTGGA
PLAG1	F: GTTAAAGCCCCGCGATTGG
	R: GCCTTGGTGCAGTCTTGTTG
U6	F: CTCGCTTCGGCAGCACATA
	R: AACGATTCACGAATTTGCGT
GAPDH	F: GATGCTGGCGCTGAGTACG
	R: GCTAAGCAGTTGGTGGTGC

*RT-qPCR* everse transcription quantitative polymerase chain reaction, *VEZF1* vascular endothelial zinc finger 1, *miR* microRNA, *PLAG1* pleomorphic adenoma gene 1, *GAPDH* glyceraldehyde-3-phosphate dehydrogenase

### Western blot analysis

Total protein was separated through radio-immunoprecipitation assay lysis buffer solution and the concentration of total protein was assessed using bicinchoninic acid kit (Beyotime Biotechnology Co., Ltd., Shanghai, China). The quantified protein lysates were loaded on 10% sodium dodecyl sulfate-polyacrylamide gel electrophoresis and then transferred onto polyvinylidene fluoride membranes (Millipore, Billerica, MA, USA), which were blocked with 5% skim milk in tris-buffered saline containing Tween-20 (TBST) at room temperature for 1 h and then incubated with primary antibodies: anti-VEZF1 (ab50970, 1:50), anti-PLAG1 (ab181457, 1:500), anti-Acyl-CoA synthetase long-chain family member 4 (ACSL4, ab155282, 1:10000), anti-glutathione peroxidase 4 (GPX4, ab125066, 1:1000), and anti-GAPDH (ab9485, 1:2500) (all from Abcam Inc., Cambridge, MA, USA) at 4℃ overnight. After that, the membranes were immersed in TBST, and incubated with secondary antibody goat anti-rabbit immunoglobulin G (IgG, ab6721, 1:2000) or rabbit anti-mouse IgG (both from Abcam) at room temperature for 1 h. Additionally, bands on membranes were developed by enhanced chemiluminescence reagent (Pierce Biotechnology, Rockford, IL, USA), and the signal was analyzed by Image-Pro Plus 6.0 software.

### Fe^2+^ content assessment

Fe^2+^ content in SK-OV-3 or A2780 cells was analyzed in accordance with the instructions of Iron Assay kit (ab83366, Abcam). Specifically, SK-OV-3 or A2780 cells were incubated with assessment buffer solution at 37℃ for 30 min, followed by 60 min of incubation with iron probes at 37℃ for 60 min. OD value at 593 nm was determined using a microplate reader (Thermo Fisher Science, Waltham, MA, USA).

### Assessment of Reactive Oxygen Species (ROS), Malondialdehyde (MDA) and Glutathione (GSH)

The levels of ROS in SK-OV-3 or A2780 cells were detected using the ROS assay kit (E004-1-1, Nanjing Jiancheng Bioengineering Institute, Nanjing, Jiangsu, China) following the manufacturer’s instructions: DCFH-DA was added to the culture medium, and cells were incubated at 37℃ for 30 min. Cells were collected after centrifugation, resuspended in PBS, and the absorbance of each tube was measured at 525 nm.

The levels of MDA in SK-OV-3 or A2780 cells were determined using the MDA assay kit (A003-1-2, Nanjing Jiancheng Bioengineering Institute) according to the manufacturer’s protocols: Reagent 1 in the kit was pre-warmed at 37℃ to accelerate dissolution until transparent; 340 mL of distilled water was added to each bottle of Reagent 2, mixed well, and stored at 4℃; the powder was dissolved in 60 mL of distilled water, heated to 90–100℃ for complete dissolution, then supplemented with distilled water to 60 mL, followed by the addition of 60 mL of glacial acetic acid and mixing well, and the prepared solution was stored in the dark at 4℃. In a centrifuge tube, 1 mL of Reagent 1 was added and mixed, followed by 3 mL of Reagent 2 and 1 mL of Reagent 3. The centrifuge tube was capped, a small hole was punctured in the cap with a needle, and the mixture was vortexed using a vortex mixer. After incubation in a 95℃ water bath for 40 min, the tube was taken out and cooled under running water, then centrifuged at 1000 g for 10 min. The supernatant was collected, and the absorbance of each tube was measured at 532 nm.

The levels of GSH in SK-OV-3 or A2780 cells were measured using the GSH assay kit (A005-1-2, Nanjing Jiancheng Bioengineering Institute) following the manufacturer’s instructions: 0.2 mL of GSH (1 mmol/L) was added to a centrifuge tube and pre-warmed in a 37℃ water bath for 5 min, then 0.1 mL of Reagent 1 was added and pre-warmed in a 37℃ water bath for another 5 min. After adding 2 mL of Reagent 2 and mixing well, the mixture was centrifuged at 1000 g for 10 min, and 1 mL of the supernatant was taken for color reaction. Subsequently, 1 mL of Reagent 3, 0.25 mL of Reagent 4, and 0.05 mL of Reagent 5 were added, mixed well, and incubated at room temperature for 15 min. The OD value of each tube was measured at 412 nm.

### Bioinformatics

The binding relation between VEZF1 and miR-545-3p promoter was predicted through Jaspar (https://jaspar.elixir.no/) [24]. Downstream target genes of miR-545-3p were analyzed through Starbase (http://starbase.sysu.edu.cn/) [25], Targetscan (http://www.targetscan.org/vert_72/) [26], miRDB (http://mirdb.org/) [27] and miRWalk (http://mirwalk.umm.uni-heidelberg.de/) databases [28]. The binding sites between miR-545-3p and PLAG1 were predicted through Starbase database.

### Dual-luciferase reporter gene assay

The synthesized miR-545-3p promoter region sequence containing VEZF1 binding sites [miR-545-3p-wild type (WT)] or that containing mutant sites [miR-545-3p-mutant type (MUT)] was respectively constructed into dual-luciferase reporter plasmids (GenePharma, Shanghai, China), which were co-transfected with oe-NC or oe-VEZF1 into SK-OV-3 cells. Similarly, PLAG1-WT (PLAG1 gene fragments containing miR-545-3p binding sites) or PLAG1-MUT (PLAG1 gene fragments containing miR-545-3p mutant sites) was respectively constructed into dual-luciferase reporter plasmids (GenePharma), which were co-transfected with miR-545-3p mimic (miR mimic) or mimic NC into SK-OV-3 cells. After 48 h, luciferase activity was determined by dual-luciferase reporter assay system (Promega, Madison, WI, USA).

### Chromatin Immunoprecipitation (ChIP) assay

ChIP assay was performed following the instructions of EZ-ChIP kit (Millipore) to verify the binding relation between VEZF1 and miR-545-3p promoter region. Briefly, SK-OV-3 cells were cross-linked with formaldehyde, subjected to ultrasound to obtain DNA fragments, which were immunoprecipitated by anti-VEZF1 antibody (Santa Cruz Biotechnology, Inc, Santa Cruz, CA, USA) or anti-IgG antibody (ab6757, Abcam). Subsequently, DNA of immunoprecipitated DNA-protein complexes was extracted, purified using fragmented DNA purification kit (Intron Biotechnology, Gyeonggi-do, Korea) and then detected by RT-qPCR, with GAPDH as the NC. Primer sequence of miR-545-3p promoter region was forward: 5′**-**TTGCCCACCCTTGGTTTAGA-3′ and reverse: 5′**-**ACACTTGCTTTCTTGGTGGGA-3′.

### Statistical analysis

SPSS 21.0 (IBM SPSS Statistics, Chicago, IL, USA) was applied for data analysis and GraphPad Prism 8.0 software (GraphPad Software Inc., San Diego, CA, USA) was employed for graphing. The results were exhibited as mean ± standard deviation. All data were inspected with normality distribution and homogeneity of variance test. Comparisons between two groups were analyzed with *t* test, and comparisons among multiple groups were analyzed with one-way or two-way analysis of variance (ANOVA). Tukey’s multiple comparisons test was used for post hoc test. The *p* value was obtained from two-tailed tests, with *p* < 0.05 indicating significant differences.

## Results

### Silencing of VEZF1 accelerates OC cell ferroptosis

**Fig. 1:**
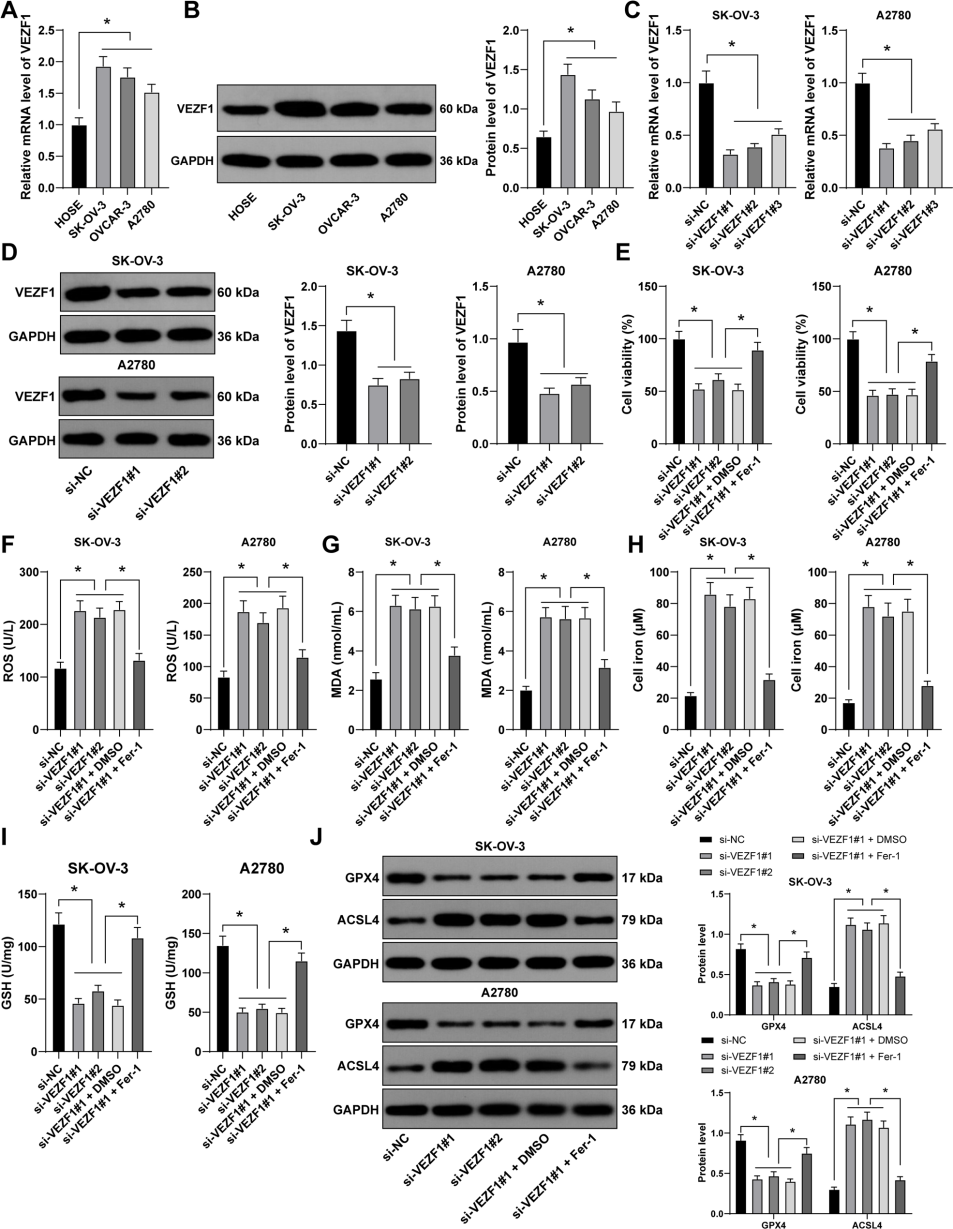
Silencing of VEZF1 accelerates OC cell ferroptosis. **A** and **B**, VEZF1 expression in HOSE cell line and OC cell lines was detected by RT-qPCR (**A**) and western blot analysis (**B**). si-VEZF1#1, si-VEZF1#2 or si-VEZF1#3 was respectively transfected into SK-OV-3 and A2780 cells, with si-NC transfection as the NC, and si-VEZF1#1 and si-VEZF1#2 were selected for the following experiments for their better transfection efficiency. **C** and **D**, VEZF1 expression in SK-OV-3 and A2780 cells was examined by RT-qPCR (**C**) and Western blot (**D**). SK-OV-3 and A2780 cells were treated with 1 mM Fer-1, with DMSO serving as the negative control, and combination experiments were conducted with si-VEZF1#1. **E**, cell proliferation was assessed by CCK-8 method. **F**-**I**, levels of ROS, MDA, Fe^2+^ and GSH in SK-OV-3 and A2780 cells were verified. **J**: The expression of GPX4 and ACSL4 in SK-OV-3 and A2780 cells was detected by Western blot. *n* = 3. Data in panels A-J were presented as mean ± standard deviation. One-way ANOVA was used to analyze the data in panels A-I and two-way ANOVA to analyze the data in panel J. Tukey’s multiple comparisons test was applied for post hoc test. *p* values were determined by two-tailed test. * *p* < 0.05

Transcription factor VEZF1 was abundantly expressed in OC [14]. To explore the role of VEZF1 in OC cells, VEZF1 expression in HOSE cell line and OC cell lines (SK-OV-3, OVCAR-3 and A2780) was examined, and the results found that VEZF1 mRNA level and protein expression were higher in OC cell lines than those in HOSE cell line (*p* < 0.05, Fig. 1A-B). Ferroptosis is a vital regulatory mechanism in a variety of malignancies, including OC [8, 29]. Subsequently, we selected SK-OV-3 and A2780 cells, which displayed the highest and lowest VEZF1 expression, for subsequent experiments. Transfection of si-VEZF1#1, si-VEZF1#2 or si-VEZF1#3 into SK-OV-3 and A2780 cells successfully downregulated the expression of VEZF1 (*p* < 0.05, Fig. 1C-D), with si-VEZF1#1 and si-VEZF1#2, the ones with better transfection efficiency, selected for the following experiments. After downregulating VEZF1 expression, cell proliferation was suppressed (*p* < 0.05, Fig. 1E). Then, levels of ROS and MDA were determined to assess oxidative stress degree, and it was noticed that levels of ROS and MDA were elevated after silencing of VEZF1 (*p* < 0.05, Fig. 1F-G). And the detection on Fe^2+^ content revealed that it was accumulated upon VEZF1 silencing (*p* < 0.01, Fig. 1H). It was reported that levels of GSH and GPX4 were negatively related to ferroptosis [30]. It was found that VEZF1 silencing resulted in inactivated GSH and GPX4 levels but increased ferroptosis-related protein ACSL4 expression (*p* < 0.05, Fig. 1I-J). Studies have demonstrated that Fer-1 can effectively inhibit ferroptosis [9, 31]. We performed combined SK-OV-3 and A2780 cells with Fer-1 and si-VEZF1#1, and the results showed that after Fer-1 treatment, the viability of SK-OV-3 and A2780 cells was increased, the levels of ROS, MDA, and Fe²⁺ were decreased, the levels of GSH and GPX4 were increased, and the expression of ACSL4 was downregulated (*p* < 0.05, Fig. 1E-J). Collectively, silencing of VEZF1 accelerated OC cell ferroptosis.

### VEZF1 transcriptionally inhibits miR-545-3p expression

**Fig. 2:**
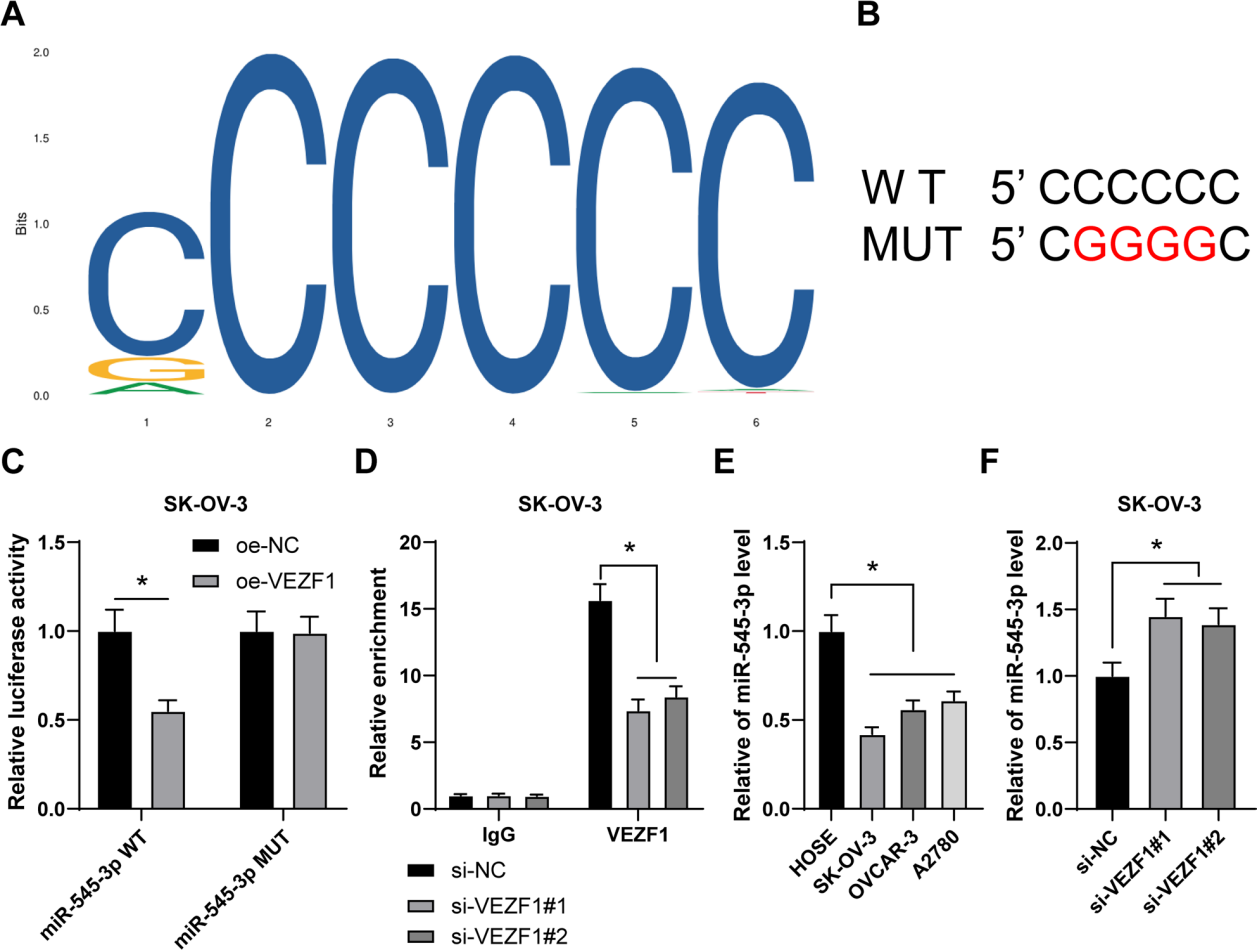
VEZF1 transcriptionally inhibits miR-545-3p expression. **A **and **B**, the binding relation between VEZF1 and miR-545-3p was predicted through JASPAR database. **C** and **D**, the binding relation between VEZF1 and miR-545-3p was verified dual-luciferase reporter gene assay (**C**) and ChIP assay (**D**). **E**, miR-545-3p expression in HOSE cell line and OC cell lines was detected by RT-qPCR. **F**, miR-545-3p expression in SK-OV-3 cells was detected by RT-qPCR. *n* = 3. Data in panels C, D, E and F were presented as mean ± standard deviation. Two-way ANOVA was used to analyze the data in panels C and D, and one-way ANOVA to analyze the data in panels E and F. Tukey’s multiple comparisons test was applied for post hoc test. *p* values were determined by two-tailed test. * *p* < 0.05

Transcription factors could modulate miRs to interfere human carcinomas, including OC [13, 32, 33]. JASPAR database predicted that VEZF1 could bind to miR-545-3p promoter region (Fig. 2A-B). miR-545-3p was downregulated in OC [34], and it catalyzed cancer cell ferroptosis to quench gastric cancer pathogenesis [35]. Hence, dual-luciferase reporter gene assay was formulated to verify the binding relation of VEZF1 to miR-545-3p (*p* < 0.05, Fig. 2C). ChIP assay revealed that VEZF1 enriched on miR-545-3p promoter, while VEZF1 silencing discouraged VEZF1 enrichment (*p* < 0.05, Fig. 2D). miR-545-3p expression was then detected, and the result demonstrated that miR-545-3p expression was lower in OC cell lines than that in HOSE cell line (*p* < 0.05, Fig. 2E), but VEZF1 silencing led to elevated miR-545-3p expression in SK-OV-3 cells (*p* < 0.05, Fig. 2F). In a word, VEZF1 could transcriptionally inhibit miR-545-3p expression.

### miR-545-3p knockdown partially reverses the effect of VEZF1 silencing on OC cell ferroptosis

**Fig. 3:**
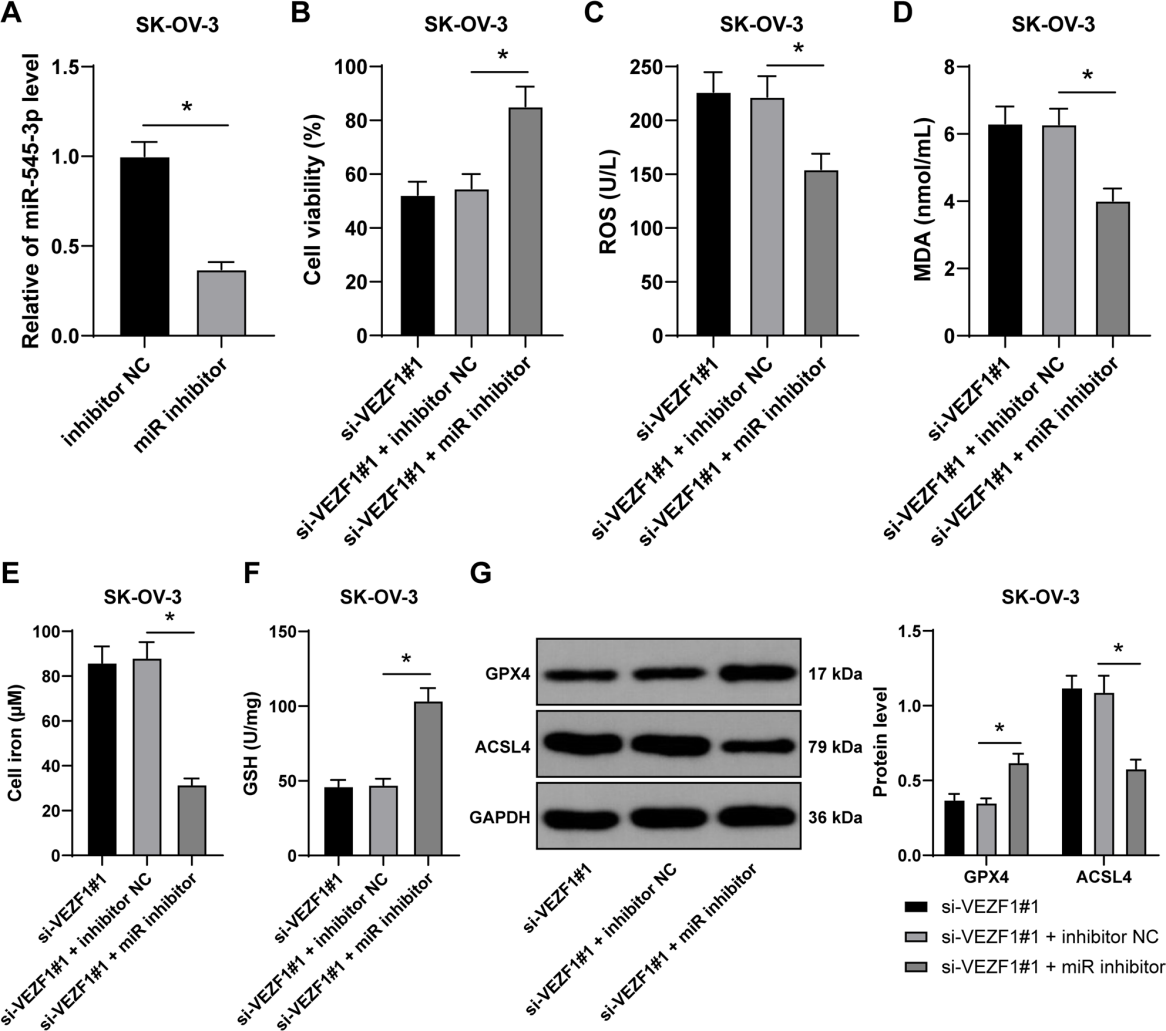
miR-545-3p knockdown partially reverses the effect of VEZF1 silencing on OC cell ferroptosis. Combined experiments were performed as miR inhibitor and si-VEZF1#1 were transfected into SK-OV-3 cells, with inhibitor NC as the control. **A**, transfection efficiency of miR-545-3p in SK-OV-3 cells was measured by RT-qPCR. **B**, cell proliferation was assessed by CCK-8 method. **C**, **D**, **E **and **F**, ROS, MDA, Fe^2+^, and GSH levels were detected. **G**, expressions of GPX4 and ACSL4 were evaluated by western blot analysis. *n* = 3. Data in panels A, B, C, D, E, F and G were presented as mean ± standard deviation. The *t* test was employed to analyze data in panel A, one-way ANOVA was used to analyze the data in panels B, C, D, E and F, and two-way ANOVA to analyze the data in panel G. Tukey’s multiple comparisons test was applied for post hoc test. *p* values were determined by two-tailed test. * *p* < 0.05

To elaborate the role of miR-545-3p in OC cell ferroptosis, combined experiments were conducted as miR inhibitor was transfected into SK-OV-3 cells to successfully downregulate miR-545-3p expression (*p* < 0.05, Fig. 3A) and si-VEZF1#1 was simultaneously transfected, and it was observed that miR-545-3p knockdown promoted cell proliferation (*p* < 0.05, Fig. 3B), declined contents of ROS and MDA (*p* < 0.05, Fig. 3C-D), decreased Fe^2+^ content (*p* < 0.05, Fig. 3E), upregulated levels of GSH and GPX4 (*p* < 0.05, Fig. 3F-G), and inhibited ACSL4 expression (*p* < 0.05, Fig. 3G), indicating that miR-545-3p knockdown could partially reverse the effect of VEZF1 silencing on OC cell ferroptosis.

### miR-545-3p targets and inhibits PLAG1 expression

**Fig. 4:**
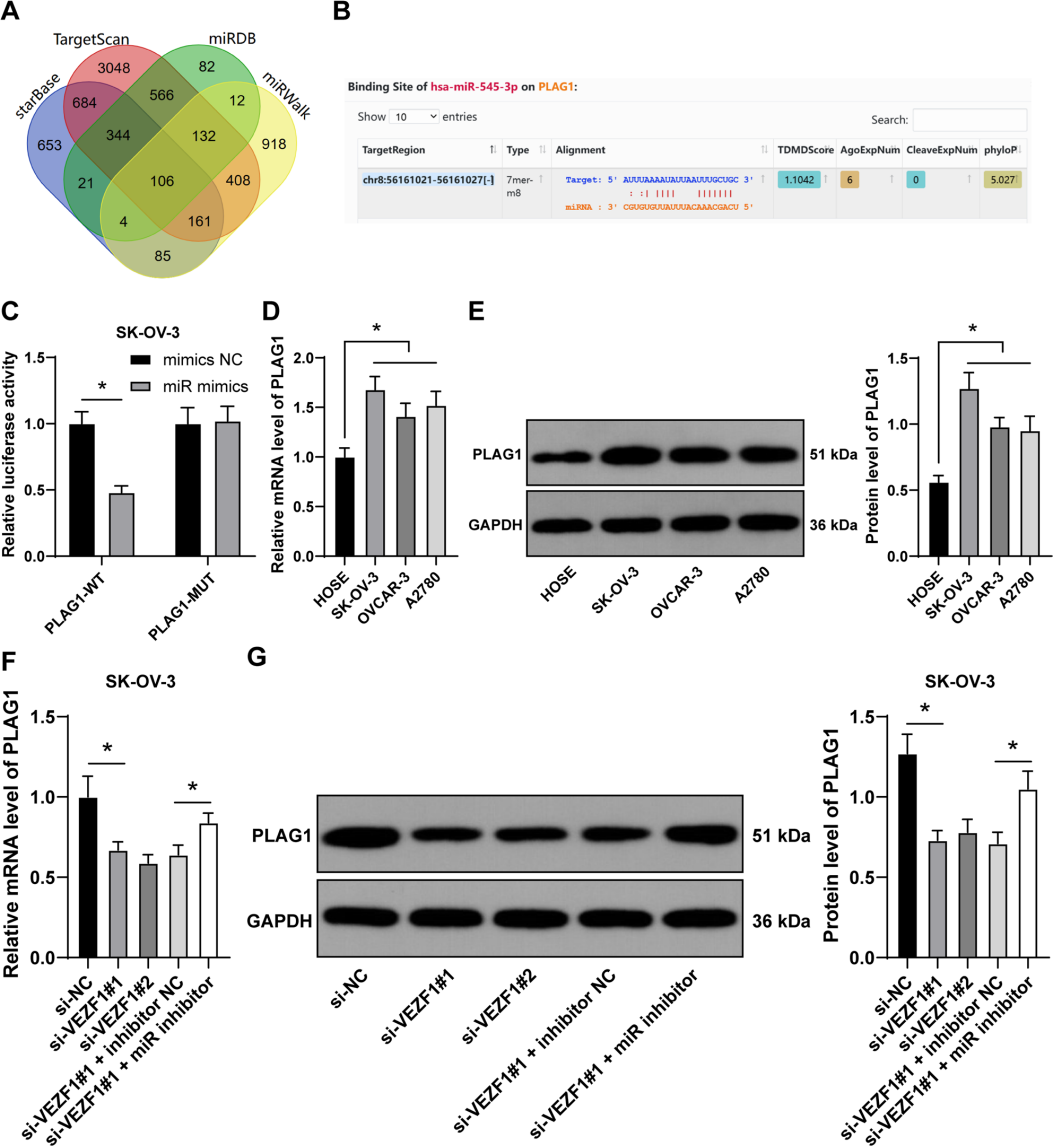
miR-545-3p targets and inhibits PLAG1 expression. **A**, StarBase, Targetscan, miRDB and miRWalk databases were employed to predict the downstream target genes of miR-545-3p, and the intersections were obtained. **B**, possible binding sites between miR-545-3p and PLAG1 were predicted through Starbase database. **C**, the binding relation between miR-545-3p and PLAG1 was verified by dual-luciferase reporter gene assay. **D **and **E**, PLAG1 expression in HOSE cell line and OC cell lines was determined by RT-qPCR (**D**) and western blot analysis (**E**). **F** and **G**, PLAG1 expression in SK-OV-3 cells was determined by RT-qPCR (**F**) and western blot analysis (**G**). *n* = 3. Data in panels **C**, **D**, **E**, **F** and **G** were presented as mean ± standard deviation. Two-way ANOVA was used to analyze the data in panel C, and one-way ANOVA to analyze the data in panels D, E, F and G. Tukey’s multiple comparisons test was applied for post hoc test. *p* values were determined by two-tailed test. * *p* < 0.05

StarBase, Targetscan, miRDB and miRWalk databases were employed to predict the downstream target genes of miR-545-3p (Fig. 4A), and PLAG1 was noticed. PLAG1 expression was activated in OC [20], and PLAG1 could suppress cancer cell ferroptosis to enhance hepatocellular carcinoma development [21]. The possible binding sites between miR-545-3p and PLAG1 were predicted through Starbase database (Fig. 4B), upon which dual-luciferase reporter gene assay was formulated to verify the binding relation of miR-545-3p to PLAG1 (*p* < 0.05, Fig. 4C). PLAG1 expression was then tested, and the result showed that OC cell lines had higher mRNA level and protein expression of PLAG1 than HOSE cell line (*p* < 0.05, Fig. 4D-E), but VEZF1 silencing led to declined PLAG1 expression in SK-OV-3 cells, while miR-545-3p knockdown led to reversed trend (*p* < 0.05, Fig. 4F-G).

### PLAG1 overexpression partially reverses the effect of VEZF1 silencing on OC cell ferroptosis

**Fig. 5:**
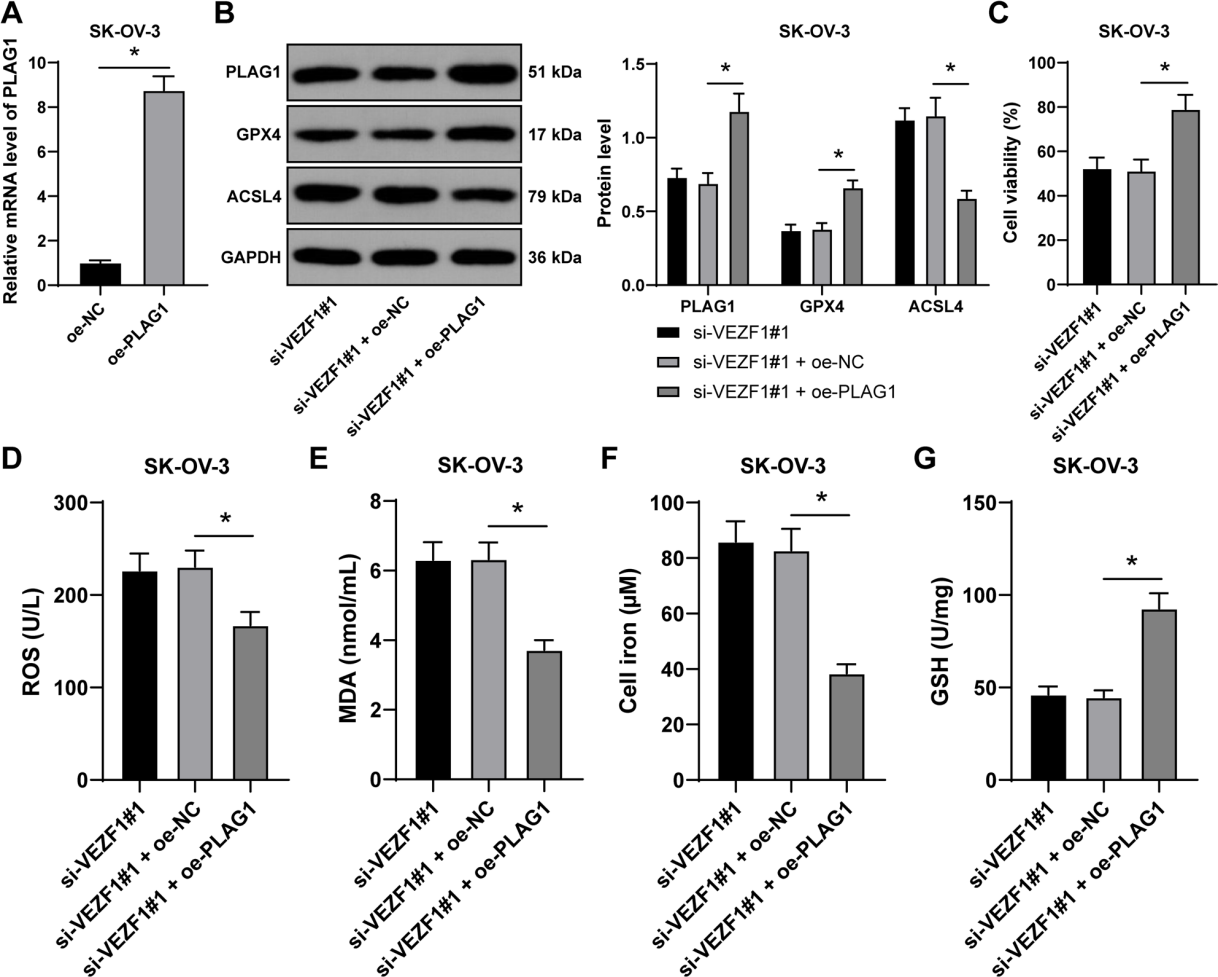
PLAG1 overexpression partially reverses the effect of VEZF1 silencing on OC cell ferroptosis. Combined experiments were performed as oe-PLAG1 and si-VEZF1#1 were transfected into SK-OV-3 cells, with oe-NC as the control. **A**, transfection efficiency of VEZF1 in SK-OV-3 cells was measured by RT-qPCR. **B**, expressions of PLAG1, GPX4 and ACSL4 were evaluated by western blot analysis. **C**, cell proliferation was assessed by CCK-8 method. **D**, **E**, **F**, and **G**, ROS, MDA, Fe^2+^, and GSH levels were detected. *n* = 3. Data in panels A, B, C, D, E, F and G were presented as mean ± standard deviation. The *t* test was employed to analyze data in panel A, one-way ANOVA was used to analyze the data in panels C, D, E, F and G, and two-way ANOVA to analyze the data in panel B. Tukey’s multiple comparisons test was applied for post hoc test. *p* values were determined by two-tailed test. * *p* < 0.05

To demonstrate the role of PLAG1 in OC cell ferroptosis, combined experiments were conducted as oe-PLAG1 was transfected into SK-OV-3 cells to successfully upregulate PLAG1 expression (*p* < 0.01, Fig. 5A-B), and si-VEZF1#1 was simultaneously transfected, and it was observed that PLAG1 overexpression was associated with promoted cell proliferation (*p* < 0.05, Fig. 5C), declined contents of ROS and MDA (*p* < 0.05, Fig. 5D-E), decreased Fe^2+^ content (*p* < 0.05, Fig. 5F), upregulated levels of GSH and GPX4 (*p* < 0.05, Fig. 5B and G), and inhibited ACSL4 expression (*p* < 0.05, Fig. 5B). In addition, PLAG1 overexpression could partially reverse the promoting effect of VEZF1 silencing on OC cell ferroptosis.

## Discussion

OC is a major reason for female death all over the world, and this malignancy could impose tremendous clinic and economic burdens as it is often diagnosed at advanced stage and it requires considerable healthcare staffs [36]. Ferroptosis is recorded as a programmed cell death characterized by Fe^2+^ content accumulation, lipid peroxidation, and glutathione degradation, and it contributes to the suppression of OC malignancy [29]. Transcription factors influence almost the whole process OC progression including OC cell initiation, pathogenesis, invasion, metastasis, and death [37]. VEZF1 is upregulated in triple negative breast cancer and it expedites cancer malignant behaviors by encouraging angiogenesis [38]. However, there are only a few published documents about the role of VEZF1 in OC, let alone the effect of VEZF1 in OC cell ferroptosis. Our experiment is hoped to explore the underlying treatment for OC with the understanding of VEZF1 and its related pathway. Eventually, we found that VEZF1 was overexpressed in OC, and it impaired OC cell ferroptosis via the miR-545-3p/PLAG1 axis.

Primarily, it was discovered in this research that VEZF1 was overexpressed in OC, while VEZF1 silencing led to elevated levels of ROS and MDA, recruited Fe^2+^ content, inactivated GSH and GPX4 levels but increased ACSL4 expression. Oxidative stress triggers different kinds of neoplasms, including OC, and is involved in cancer cell viability and mobility as well as declined susceptibility to therapies, and increased level of oxidative stress induces cancer cell death and dysfunction [39]. Intriguingly, high contents of Fe^2+^, ROS and MDA functioned as a signal of accelerated ferroptosis in solid tumors [40]. VEZF1 reduced ferroptosis and induced cellular damages with the involvement of GPX4 expression [18]. ACSL4 could manipulate lipid metabolic activities, and when ACSL4 expression was upregulated, OC proliferation was alleviated and ferroptosis was promoted as manifested by increased levels of Fe^2+^, ROS and MDA and decreased GSH level [41]. Collectively, silencing of VEZF1 accelerated OC cell ferroptosis.

Transcription factors and miRs could form a feedback loop to modulate cancerous transformation, detection and alleviation [42]. Likewise, VEZF1 was negatively associated with miR-19b-1 to mediate breast cancer metastasis and viability [43]. Although no studies have identified VEZF1 as a transcriptional repressor regulating the expression of downstream genes in OC to date, a previous report has demonstrated that VEZF1 transcriptionally represses the expression of the anti-angiogenic factor Cited2 in endothelial cells [44]. The present study reveals that VEZF1 transcriptionally inhibited miR-545-3p in OC cells. miRs are a group of non-coding RNAs that play coordinated role in OC cell pathogenesis, proliferation, impairment, aggressiveness and death, and their dysregulation is involved in variances of OC epigenetic features and biological heterogeneity [45]. miR-545-3p was deemed as a benign indicator in various carcinomas as it could expedite cell failure and death [46–48]. Taken together, to figure out the role of miR-545-3p in OC cell ferroptosis, miR-545-3p expression was downregulated, and miR-545-3p knockdown promoted cell proliferation, declined contents of ROS and MDA, decreased Fe^2+^ content, upregulated levels of GSH and GPX4, and inhibited ACSL4 expression. When miR-545-3p was abundantly expressed in OC, it decelerated cancer initiation and growth, enhanced cell vulnerability to therapy, encouraged cell death and eventually led to a favorable overall survival consequence [49]. Besides, miR-545-3p improved ferroptosis and conquered cell growth in thyroid cancer with the involvement of elevated ROS, MDA, and Fe^2+^ contents [35], further validating the beneficial effect of miR-545-3p on OC cell ferroptosis.

miR-545-3p could sabotage cancer progression by directly targeting and inhibiting its downstream oncogenic target genes [50, 51]. We have predicted that PLAG1 could be a promising target of miR-545-3p. The crosstalk between PLAG1 and miRs regulates different cellular activities in a wide range of human carcinomas [52, 53]. PLAGL2 knockout was related to exacerbated OC cell proliferation and encouraged cell death [54]. But the role of PLAG1 in OC cell ferroptosis is less clear. To extensively research the mechanism of PLAG1 in OC cell ferroptosis, PLAG1 was upregulated, after which cell proliferation was promoted, contents of Fe^2+^, ROS and MDA were declined, levels of GSH and GPX4 were upregulated, and ACSL4 expression was inhibited. Juma et al. have documented that PLAG1 is a famous tumor promoter by inducing malignant proliferation events [55]. PLAG1 activated GPX4 expression to retard lipid peroxide activity as manifested by downregulate levels of Fe^2+^, ROS and MDA, thereby hindering cancer cell ferroptosis [21], illustrating that PLAG1 disrupted OC cell ferroptosis.

Collectively, we found that VEZF1 was overexpressed in OC and it limited OC cell ferroptosis by transcriptionally inhibiting miR-545-3p and elevating PLAG1 expression. These results are prospective in promoting OC therapy. Nevertheless, there are several limitations. We only investigated the molecular mechanism of ferroptosis in OC. Changes in the intracellular levels of ROS, MDA, Fe²⁺, GSH, GPX4, and ACSL4 detected in this study indicated the occurrence of ferroptosis in cells, and treatment with the ferroptosis inhibitor Fer-1 reversed all the aforementioned indicators. However, ferroptosis is accompanied by oxidative stress, and apoptosis, pyroptosis, or necroptosis may occur concomitantly with ferroptosis in the treatment of OC [8, 56, 57]. Further investigations are therefore required to explore the role of this mechanism in other forms of cell death in future research. Besides, apart from PLAG1, other downstream target genes of PLAG1 should also be investigated to further certify our results, and downstream mechanism of PLAG1 requires further research. Additionally, although our findings demonstrate that VEZF1 acts as a transcriptional repressor of miR-545-3p, we did not examine whether this repression involves histone modifications (e.g., acetylation or methylation) or recruitment of co-repressors. Prior research indicates that VEZF1 can modulate gene expression through epigenetic mechanisms, such as altering histone H3K27 acetylation levels at target promoters or influencing DNA methylation via effects on methyltransferases [58–61]. The absence of such data in our study limits the depth of our mechanistic insights, and future investigations should incorporate assays for histone modifications and co-repressor interactions to strengthen the evidence for VEZF1’s role in this axis. In the future, we will continue to seek the potential mechanism of VEZF1 in pyroptosis or apoptosis via the miR-545-3p/PLAG1 axis, with other downstream target genes of miR-545-3p included, and explore the downstream mechanisms of PLAG1, to provide a novel theoretical basis for OC prevention.

## Supplementary Information

Supplementary Material 1.

Supplementary Material 2.

Supplementary Material 3.

Supplementary Material 4.

Supplementary Material 5.

Supplementary Material 6.

Supplementary Material 7.

Supplementary Material 8.

Supplementary Material 9.

Supplementary Material 10.

Supplementary Material 11.

Supplementary Material 12.

Supplementary Material 13.

Supplementary Material 14.

Supplementary Material 15.

Supplementary Material 16.

Supplementary Material 17.

Supplementary Material 18.

## Data Availability

The original contributions presented in the study are included in the article.
