# Supplementary figures and images for: VEZF1 inhibits ovarian cancer cell ferroptosis and acts as an oncogene via the miR-545-3p/PLAG1 axis
Source: Hereditas. 2026 Apr 13;163:65. doi: 10.1186/s41065-026-00672-z (PMC13188589; doi:10.1186/s41065-026-00672-z)

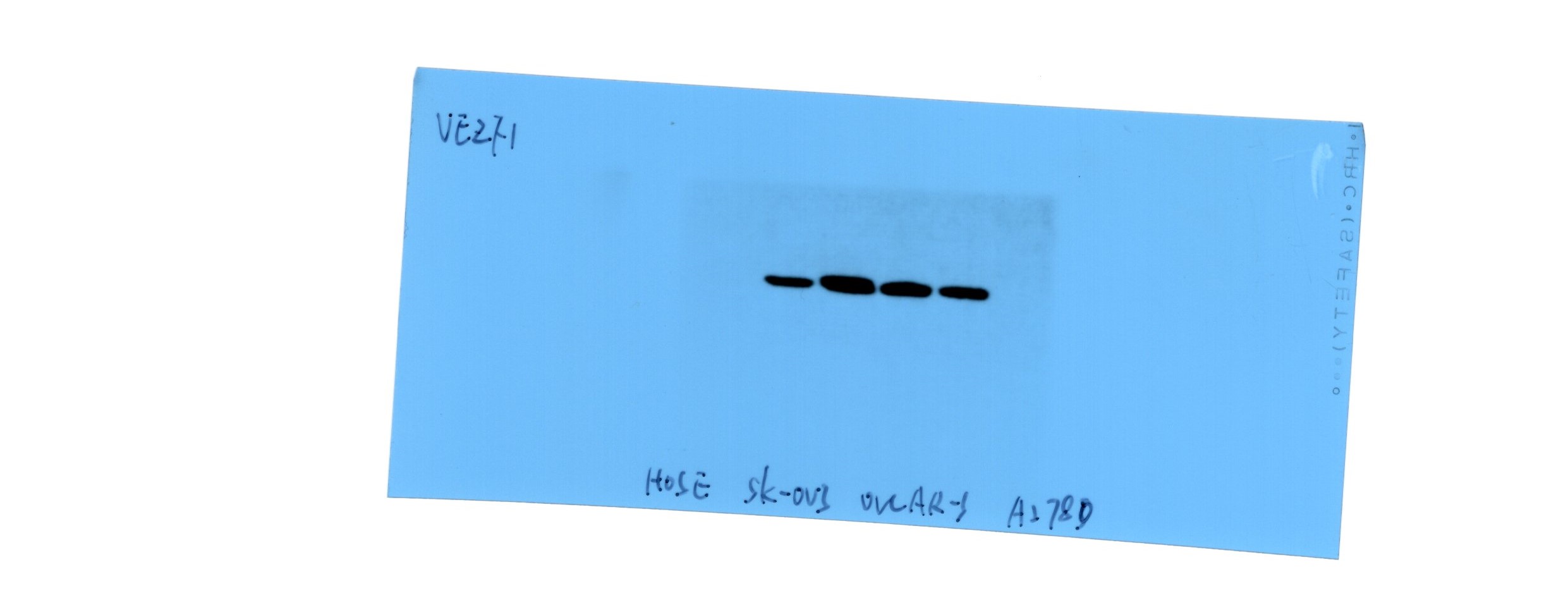

Supplement: Supplementary file 1 — Supplementary Material 1. [file 41065_2026_672_MOESM1_ESM.jpg]

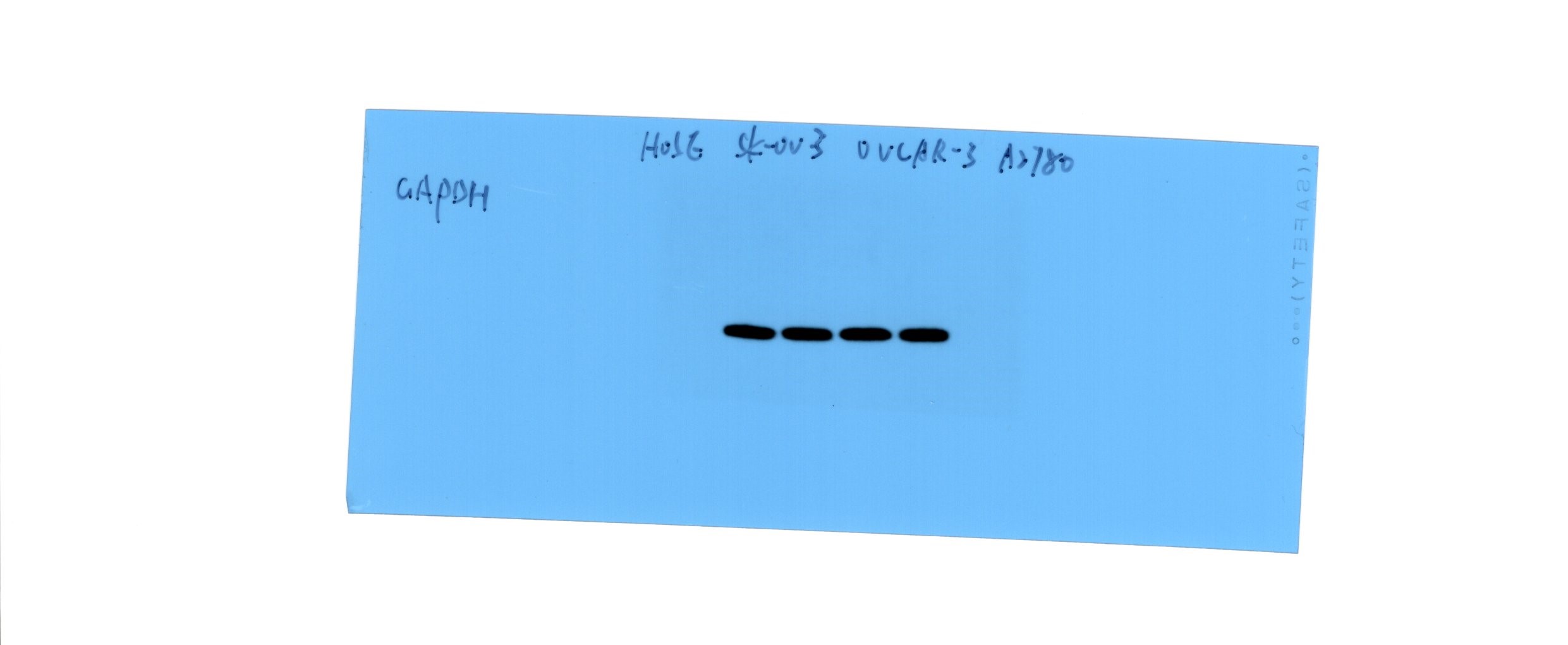

Supplement: Supplementary file 2 — Supplementary Material 2. [file 41065_2026_672_MOESM2_ESM.jpg]

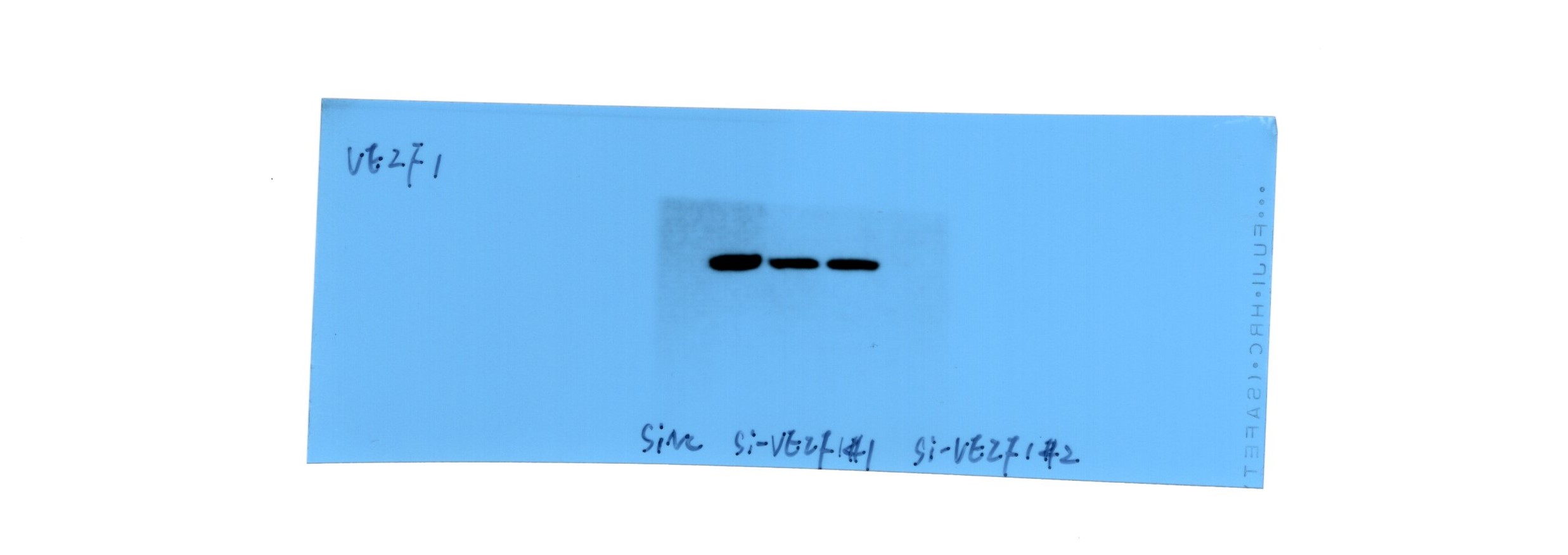

Supplement: Supplementary file 3 — Supplementary Material 3. [file 41065_2026_672_MOESM3_ESM.jpg]

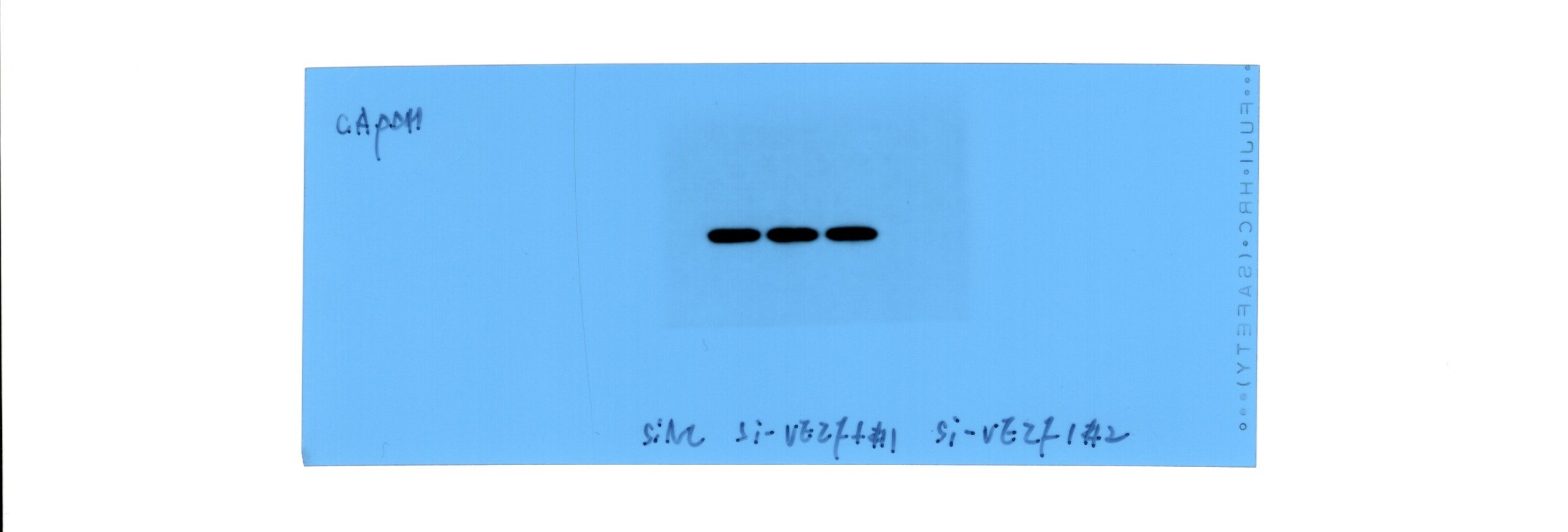

Supplement: Supplementary file 4 — Supplementary Material 4. [file 41065_2026_672_MOESM4_ESM.jpg]

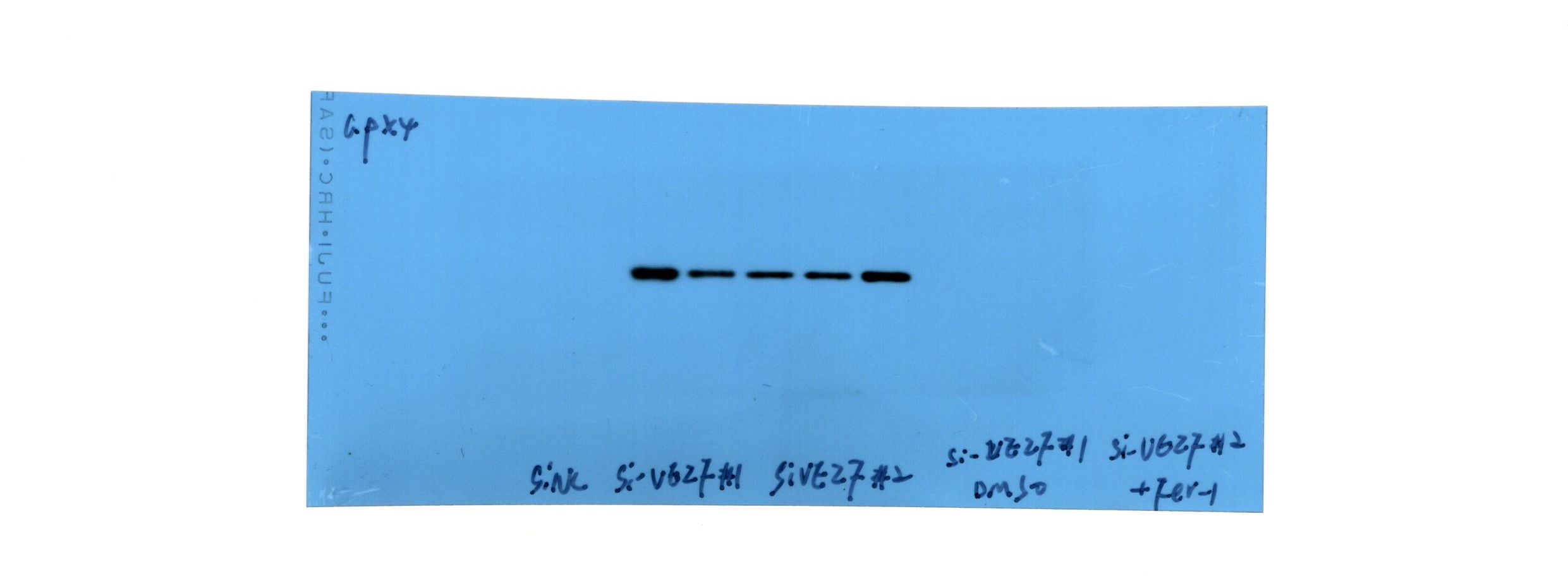

Supplement: Supplementary file 5 — Supplementary Material 5. [file 41065_2026_672_MOESM5_ESM.jpg]

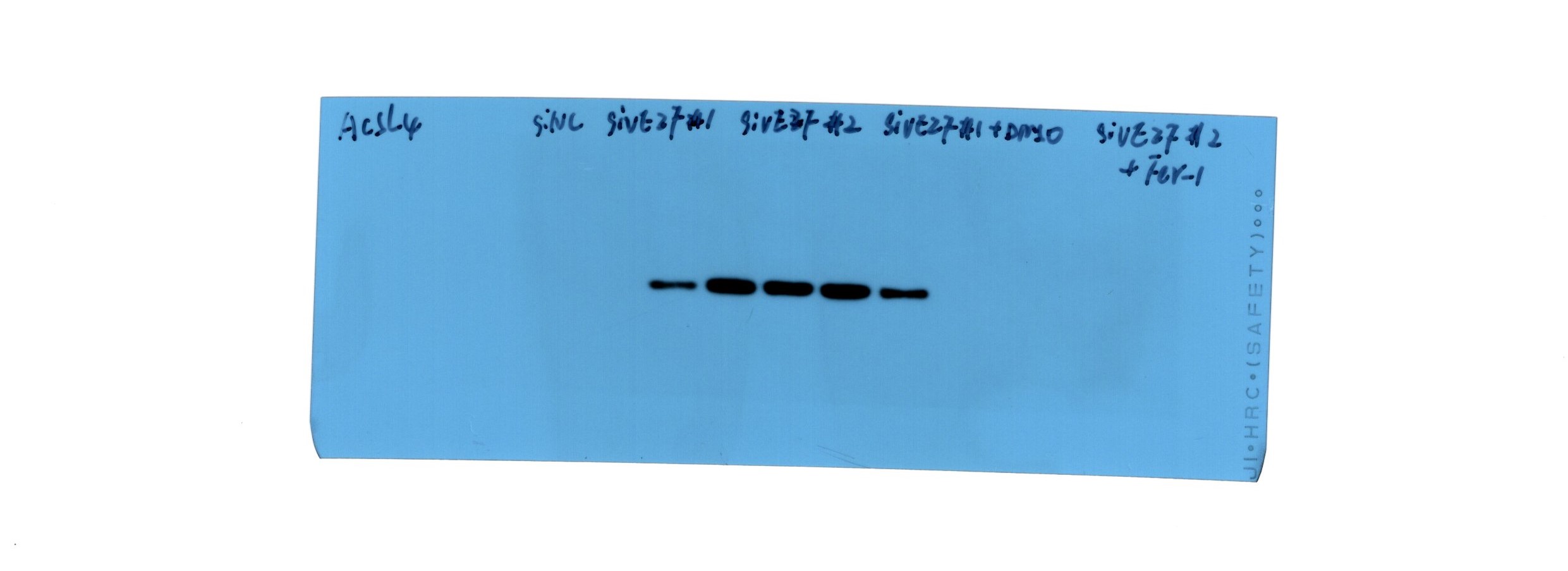

Supplement: Supplementary file 6 — Supplementary Material 6. [file 41065_2026_672_MOESM6_ESM.jpg]

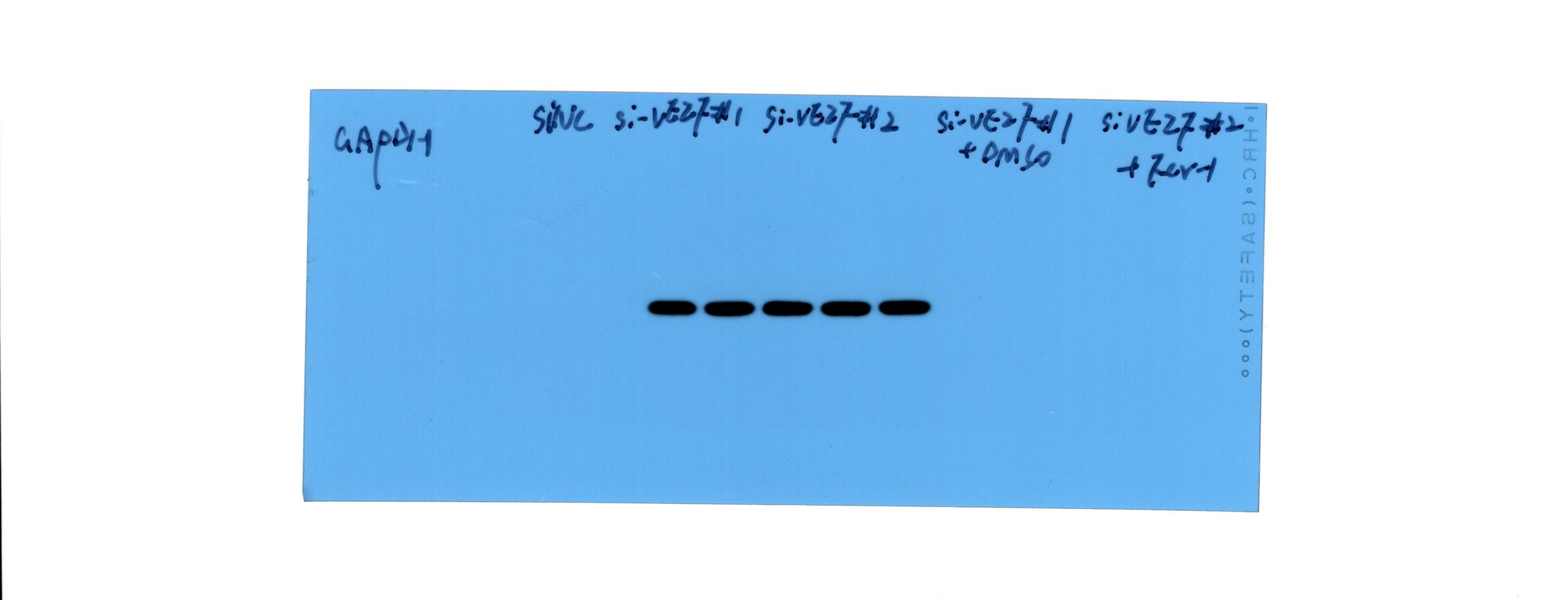

Supplement: Supplementary file 7 — Supplementary Material 7. [file 41065_2026_672_MOESM7_ESM.jpg]

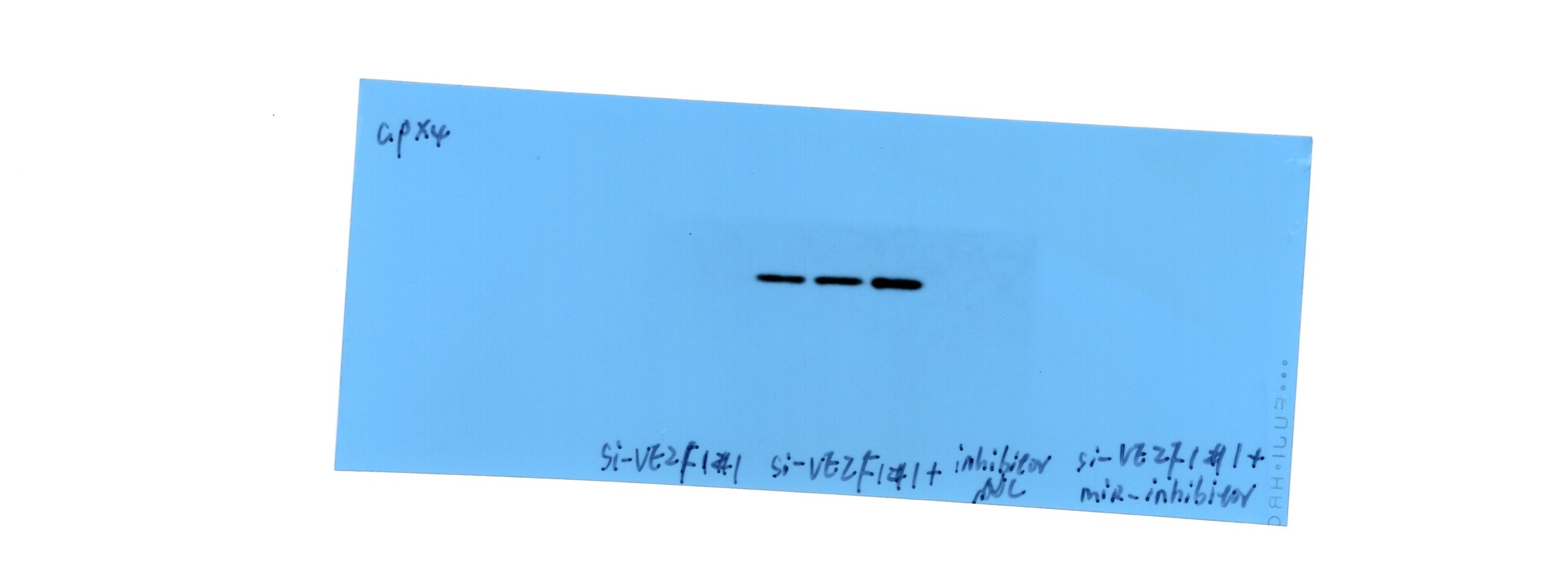

Supplement: Supplementary file 8 — Supplementary Material 8. [file 41065_2026_672_MOESM8_ESM.jpg]

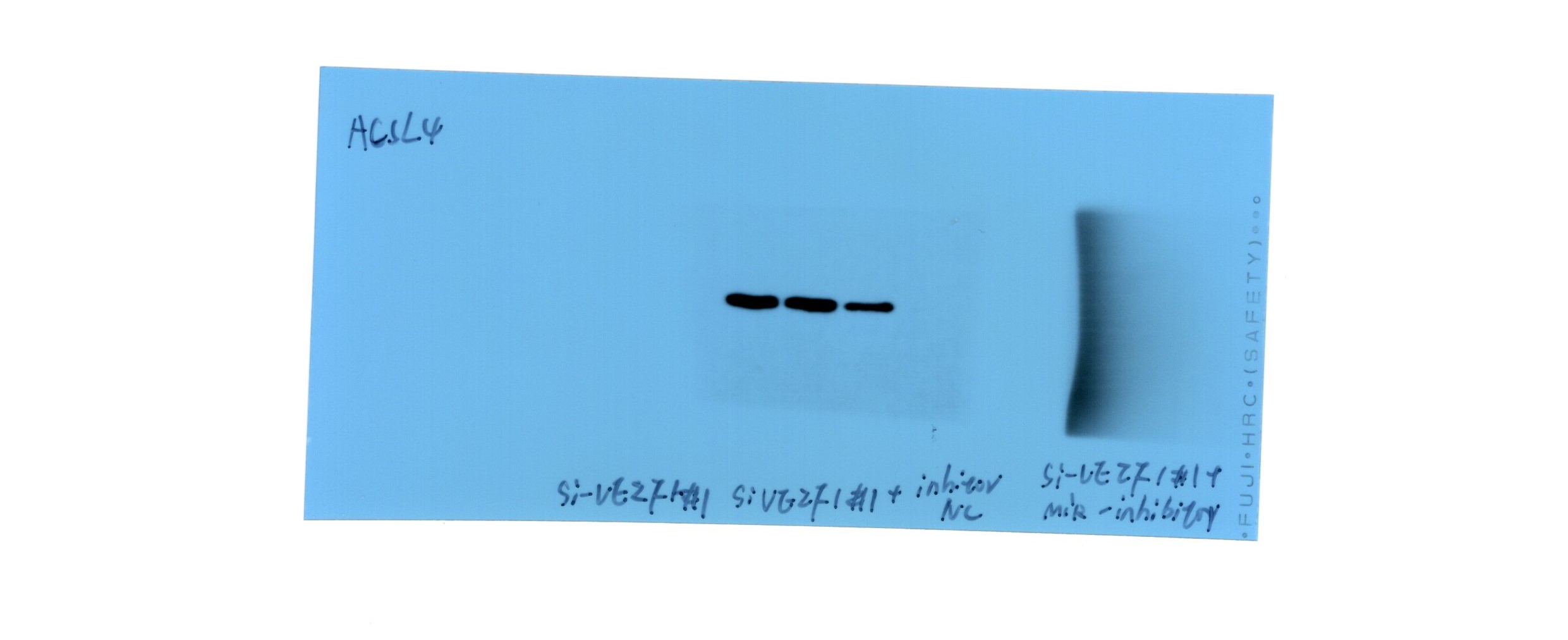

Supplement: Supplementary file 9 — Supplementary Material 9. [file 41065_2026_672_MOESM9_ESM.jpg]

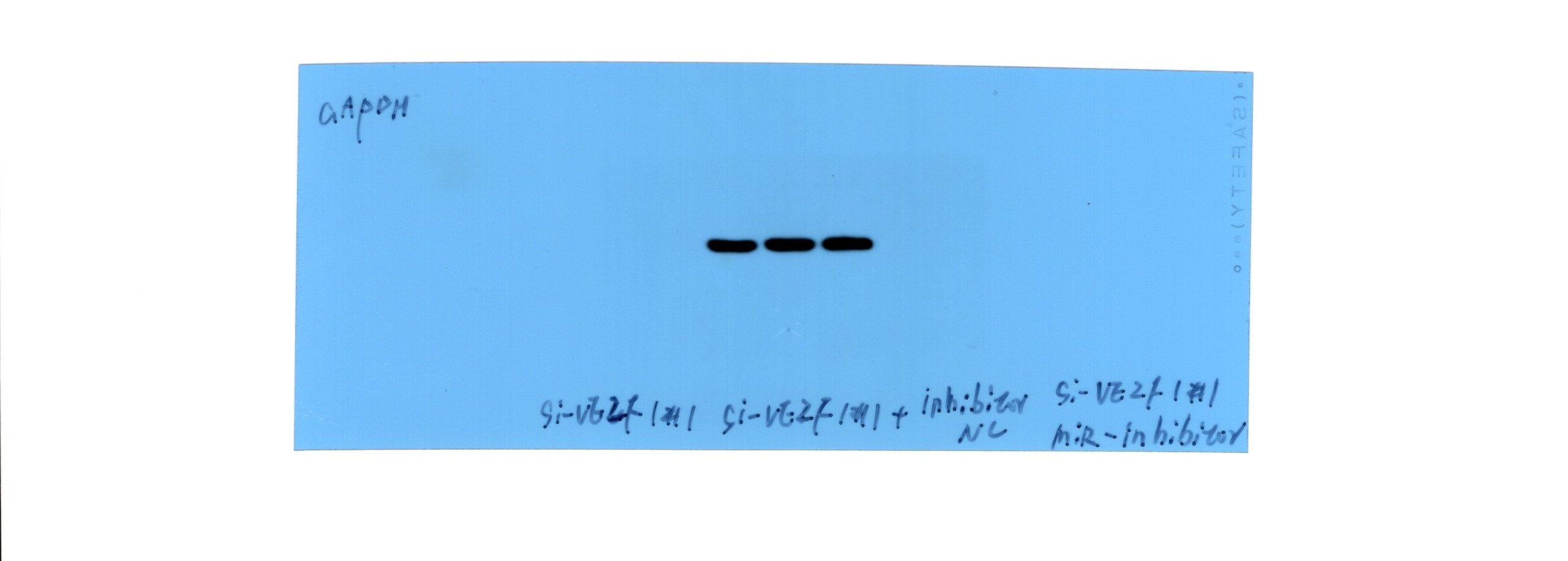

Supplement: Supplementary file 10 — Supplementary Material 10. [file 41065_2026_672_MOESM10_ESM.jpg]

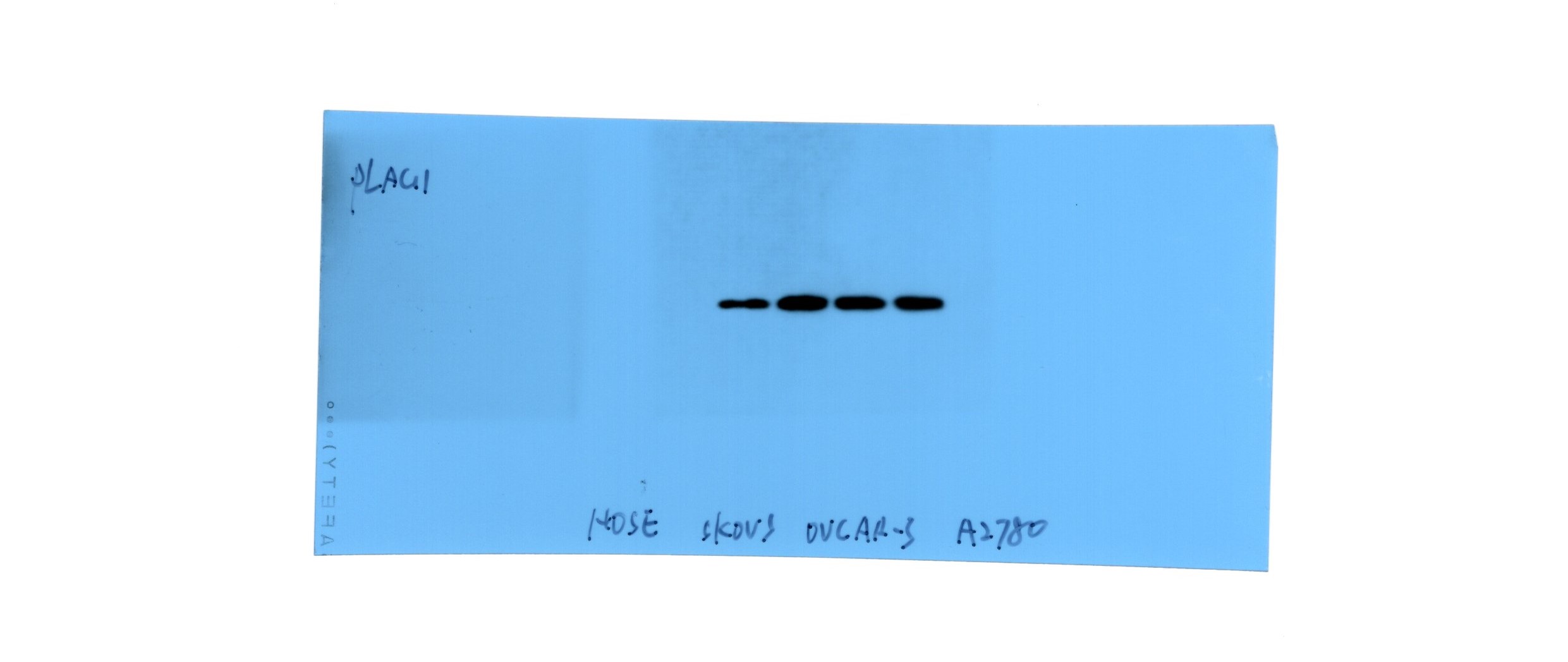

Supplement: Supplementary file 11 — Supplementary Material 11. [file 41065_2026_672_MOESM11_ESM.jpg]

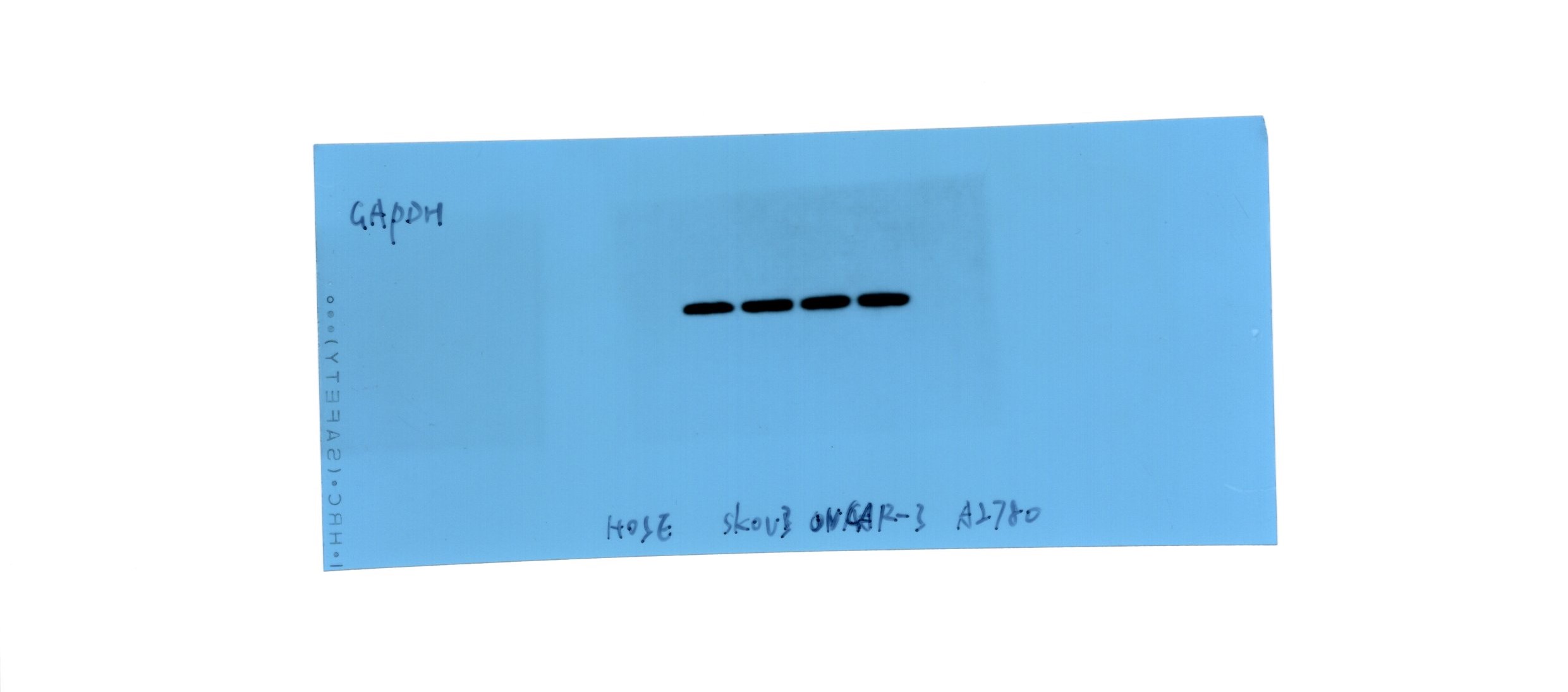

Supplement: Supplementary file 12 — Supplementary Material 12. [file 41065_2026_672_MOESM12_ESM.jpg]

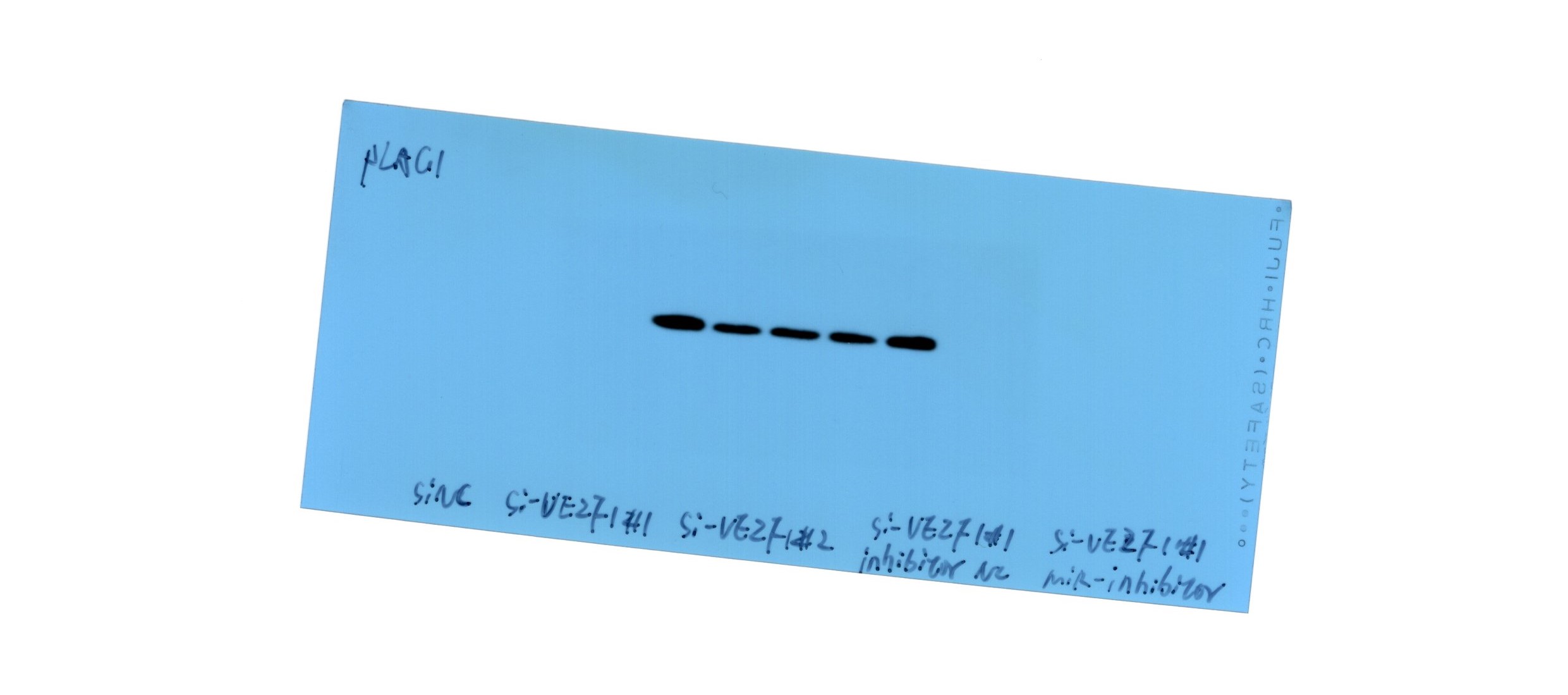

Supplement: Supplementary file 13 — Supplementary Material 13. [file 41065_2026_672_MOESM13_ESM.jpg]

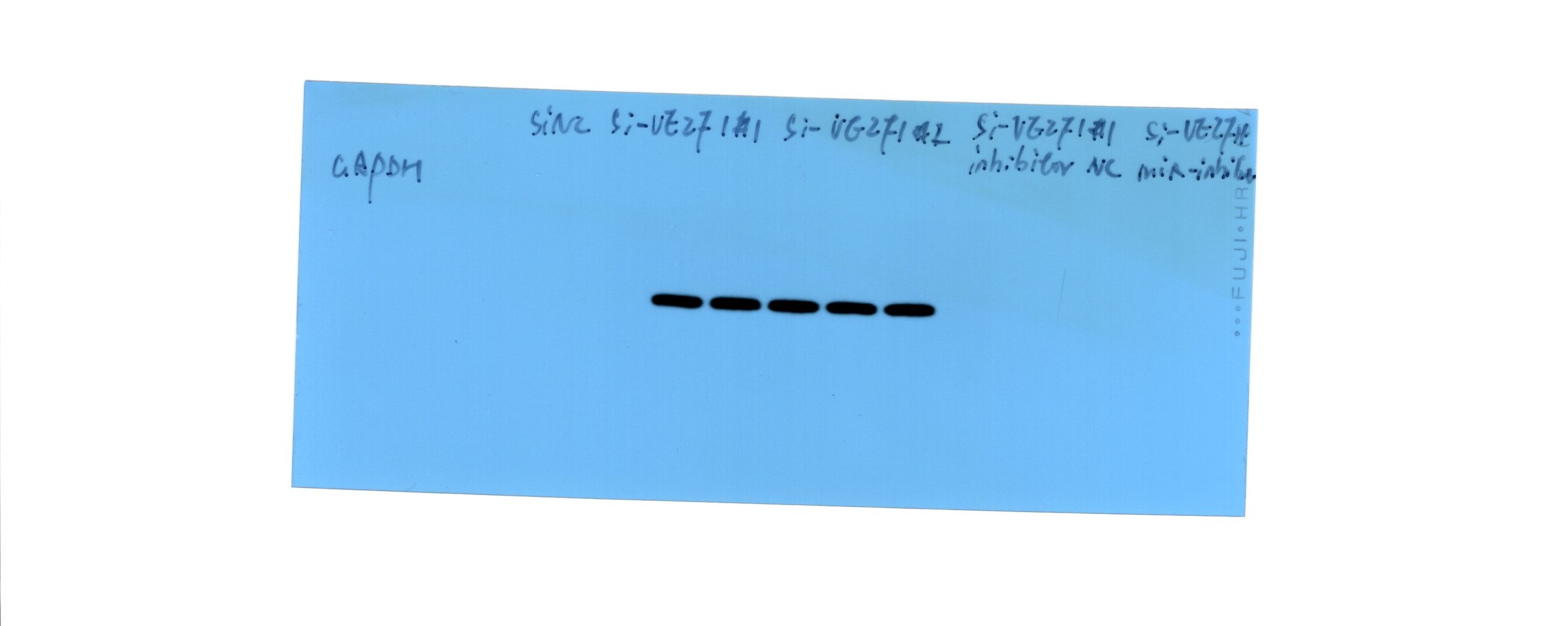

Supplement: Supplementary file 14 — Supplementary Material 14. [file 41065_2026_672_MOESM14_ESM.jpg]

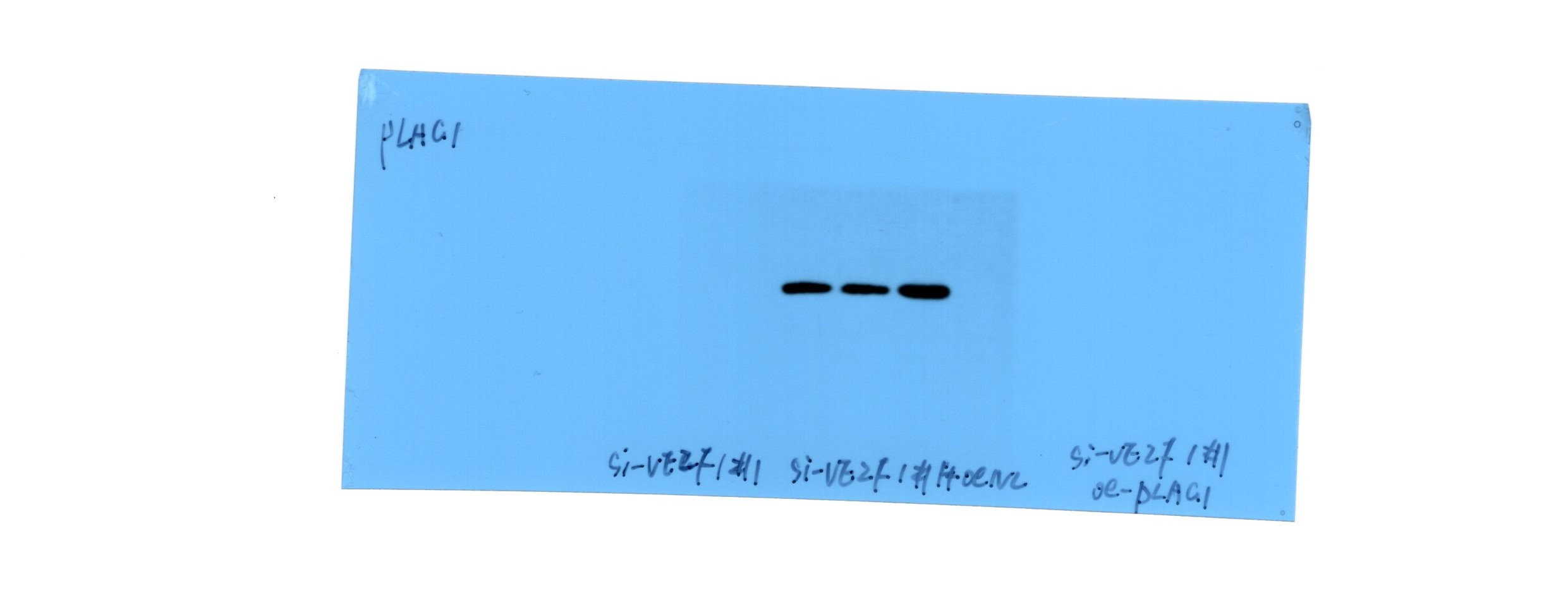

Supplement: Supplementary file 15 — Supplementary Material 15. [file 41065_2026_672_MOESM15_ESM.jpg]

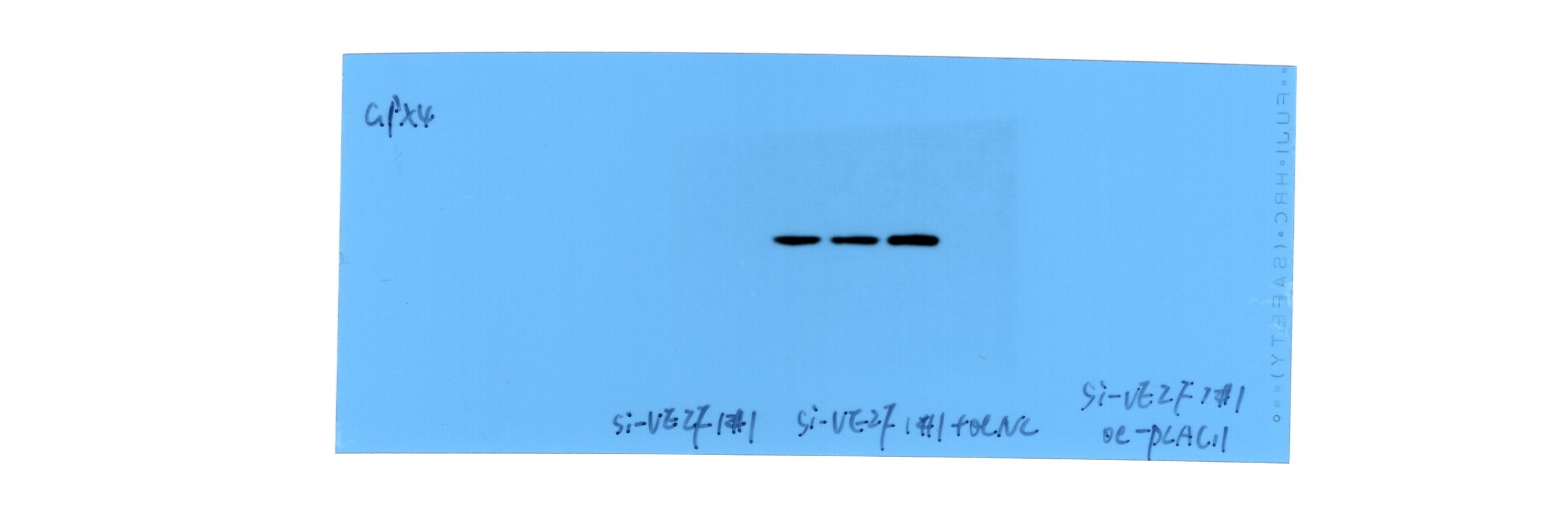

Supplement: Supplementary file 16 — Supplementary Material 16. [file 41065_2026_672_MOESM16_ESM.jpg]

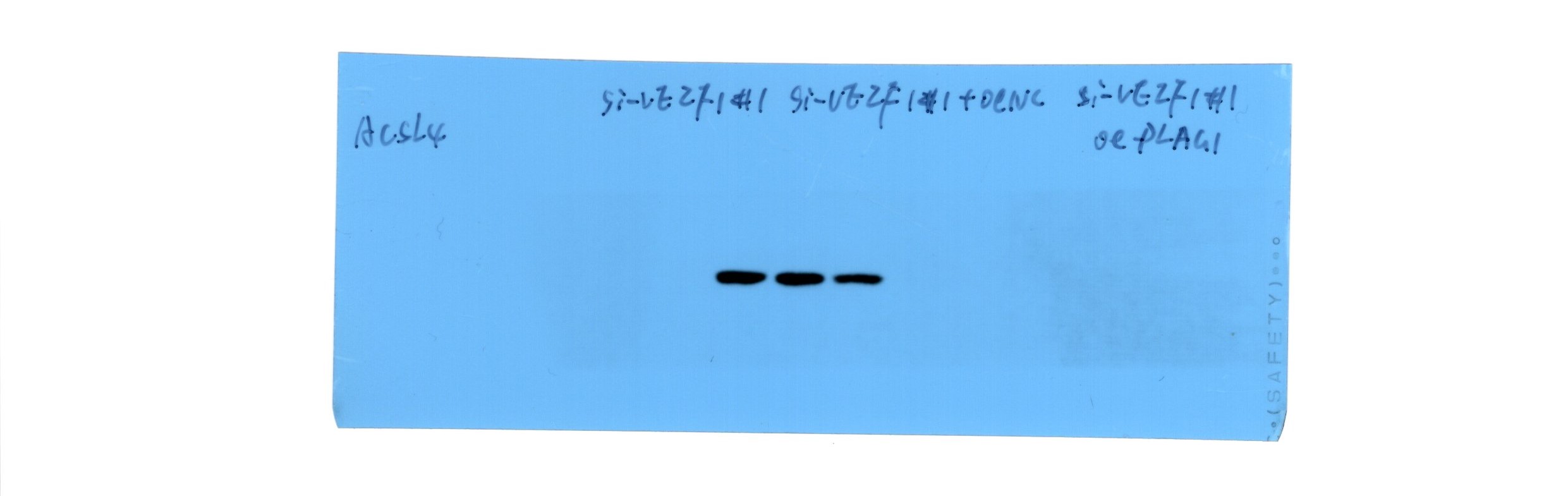

Supplement: Supplementary file 17 — Supplementary Material 17. [file 41065_2026_672_MOESM17_ESM.jpg]

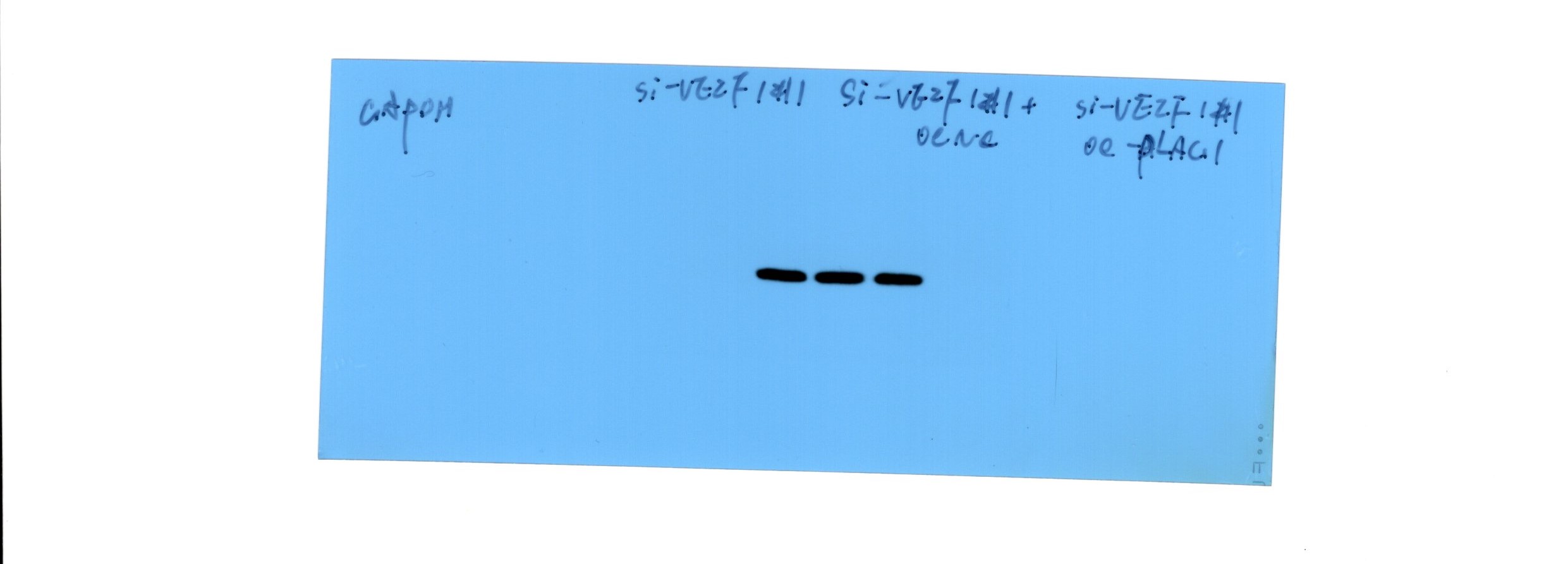

Supplement: Supplementary file 18 — Supplementary Material 18. [file 41065_2026_672_MOESM18_ESM.jpg]
